# Ultra-fast synthesis of three-dimensional porous Cu/Zn heterostructures for enhanced carbon dioxide electroreduction†

**DOI:** 10.1039/d3sc03317a

**Published:** 2023-10-04

**Authors:** Shuaiqiang Jia, Qinggong Zhu, Shitao Han, Jianxin Zhai, Mengke Dong, Wei Xia, Xueqing Xing, Haihong Wu, Mingyuan He, Buxing Han

**Affiliations:** a Shanghai Key Laboratory of Green Chemistry and Chemical Processes, School of Chemistry and Molecular Engineering, East China Normal University Shanghai 200062 China hhwu@chem.ecnu.edu.cn hanbx@iccas.ac.cn; b Beijing National Laboratory for Molecular Sciences, Key Laboratory of Colloid and Interface and Thermodynamics, Center for Carbon Neutral Chemistry, Institute of Chemistry, Chinese Academy of Sciences Beijing 100190 China qgzhu@iccas.ac.cn; c School of Chemistry, University of Chinese Academy of Sciences Beijing 100049 China; d Beijing Synchrotron Radiation Facility, Institute of High Energy Physics, Chinese Academy of Sciences Beijing 100049 China; e Institute of Eco-Chongming 20 Cuiniao Road, Chenjia Town, Chongming District Shanghai 202162 China

## Abstract

The construction of metal hetero-interfaces has great potential in the application of electro-catalytic carbon dioxide reduction (ECR). Herein, we report a fast, efficient, and simple electrodeposition strategy for synthesizing three-dimensional (3D) porous Cu/Zn heterostructures using the hydrogen bubble template method. When the deposition was carried out at −1.0 A for 30 s, the obtained 3D porous Cu/Zn heterostructures on carbon paper (CP) demonstrated a nearly 100% CO faradaic efficiency (FE) with a high partial current density of 91.8 mA cm^−2^ at −2.1 V *vs.* Ag/Ag^+^ in the mixed electrolyte of ionic liquids/acetonitrile in an H-type cell. In particular, the partial current density of CO could reach 165.5 mA cm^−2^ and the FE of CO could remain as high as 94.3% at −2.5 V *vs.* Ag/Ag^+^. The current density is much higher than most reported to date in an H-type cell (Table S1). Experimental and density functional theory (DFT) calculations reveal that the outstanding electrocatalytic performance of the electrode can be ascribed to the formation of 3D porous Cu/Zn heterostructures, in which the porous and self-supported architecture facilitates diffusion and the Cu/Zn heterostructures can reduce the energy barrier for ECR to CO.

## Abstract 2

The excellent performance of porous Cu/Zn electrodes can be attributed to rapid mass/ion diffusion, and the electronic effect between Zn and Cu lowers the energy barrier for *COOH formation on the active sites, facilitating CO generation.

## Graphical abstract

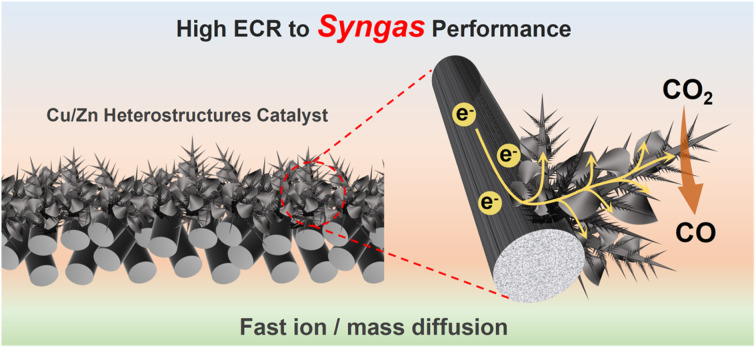

## Introduction

With rapid industrialization and economic development, the over-consumption of fossil fuels has led to an increasing accumulation of CO_2_ in the atmosphere, resulting in serious environmental problems and energy crises.^1,2^ Electrocatalytic CO_2_ reduction (ECR) has emerged as one of the attractive strategies for closing the broken carbon cycle and generating value-added fuels and chemicals. Among the reduction products, the ECR to CO is currently one of the most promising practices due to its relatively high selectivity and appreciable current density, as well as the facile separation of gas products from liquid electrolytes.^3–5^ Therefore, the fabrication of electrodes with high selectivity and high mass/ion transfer is the most important for achieving high CO selectivity.^6–8^ As a two-electron, two-proton reaction process, CO_2_ molecules are first adsorbed on the catalyst surface and then reduced to *COOH intermediates through a cooperative proton–electron transfer (CPET) process. Then, another H^+^ ion and an e^−^ ion attack *COOH to form H_2_O and *CO, and finally, *CO is desorbed from the electrode surface to form CO. It is found that the initial step is inhibited by the weak binding between the catalyst and *COOH. The desorption of CO from the electrode surface is also hindered by the strong *CO binding. These two steps are considered as rate-limiting steps in the reduction process.^9–11^ Although many noble metals have shown their ability to improve the catalytic activity of ECR to CO, their limited reserves and high prices limit their large-scale industrial application.^12^ In addition, the low concentration of CO_2_ and slow diffusivity to the catalyst, as well as sluggish reaction kinetic often lead to low conversion efficiency and poor selectivity.^13,14^ In particular, the design and preparation of low-cost and highly efficient electrodes to achieve high CO selectivity are very interesting.^15,16^

In the field of ECR, there has been growing interest in the use of heterostructure catalysts, which refer to the hetero-interfaces between multiple solid-state materials in a hybrid or composite.^17,18^ Compared with single-component metals, the chemical composition and the electronic structure of heterostructure catalysts can be tuned, which affords interesting performance in ECR.^19–21^ Recently, Cao *et al.* reported a porphyrin-based covalent organic framework (COF) containing donor–acceptor Co-based heterostructures for ECR. The combination of strong charge transfer properties and abundant active sites in the heterostructures enhanced the catalytic performance of ECR to CO with a high faradaic efficiency (FE) of 91.4% and a partial current density of 7.28 mA cm^−2^.^22^ In another study, Yuan *et al.* also found that an Ag/MnO_2_ heterostructure catalyst could provide unique interfacial properties, which presents superior activity and selectivity to CO compared to pure Ag nanoparticles.^23^ Therefore, there is general agreement that hetero-interfaces between metals can tune the adsorption energy and concentration of intermediates, thus breaking the scaling relationship of single-component catalysts and improving the selectivity for the desired product.^24–26^

In this work, we propose a hydrogen bubble template approach for ultra-fast synthesis of three-dimensional (3D) porous Cu/Zn heterostructures. We discovered that the electrode had outstanding performance, in which the porous and self-supported architecture facilitates fast mass/ion diffusion and the electronic effect between Zn and Cu lowers the energy barrier for *COOH formation on Zn sites, promoting CO production. Under optimum conditions, the 3D porous Cu/Zn heterostructures demonstrated a 99.5% CO FE with a high partial current density of 91.8 mA cm^−2^ at −2.1 V *vs.* Ag/Ag^+^ in the mixed electrolyte of ionic liquids/acetonitrile (MeCN). In particular, the partial current density of CO could reach 165.5 mA cm^−2^ and the FE of CO could remain as high as 94.3% at −2.5 V *vs.* Ag/Ag^+^ in a typical H-type cell.

## Results and discussion

**Fig. 1:**
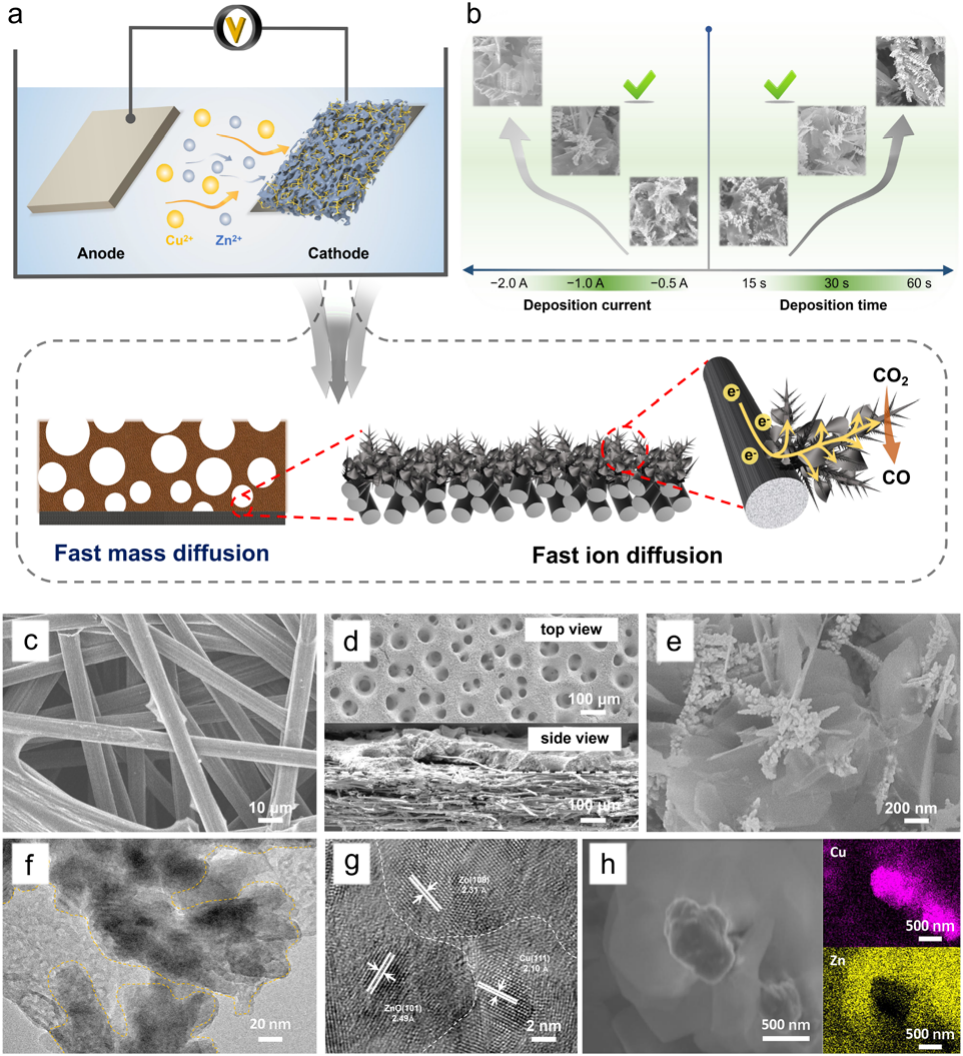
(a) Schematic illustration of the process to prepare 3D porous Cu/Zn heterostructures. (b) The effect of deposition conditions on the formation of Cu/Zn heterostructures. (c) SEM image of pure carbon paper. (d) Top view and side view images of the Cu/Zn-CP-1-30 electrode. (e) SEM image of the Cu/Zn-CP-1-30 electrode at high magnification. (f) TEM image and (g) HR-TEM image of the Cu/Zn-CP-1-30 electrode. (h) STEM image and EDS elemental mappings of the Cu/Zn-CP-1-30 electrode.

3D porous Cu/Zn heterostructures were prepared using a dynamic hydrogen bubble as a template. Scheme S1† illustrates the dynamic templating function of the hydrogen bubbles. They were formed accompanied by metal deposition, which rapidly undergoes the process of formation–condensation–growth–release, displacing the solution and inducing the formation of porous metal films. The deposition was completed in a single step when the bubbles disappeared with the interruption of the current. The bubble behavior critically depends upon various parameters like time and current density, which in turn modulate the morphology of the film.^13,27^ In a typical electrodeposition process, various Cu/Zn heterostructures (denoted as Cu_*m*_/Zn_*n*_-CP-*x-y*; *m*/*n* : [Cu^2+^]/[Zn^2+^] ratios, *x*: electrodeposition current density (A cm^−2^), *y*: electrodeposition time (s)) were obtained at different deposition current density and time. The as-prepared Cu/Zn heterostructures have beneficial physical features and facilitate ECR kinetics due to rapid mass/ion transportation (Fig. 1a).

When the deposition was carried out at −1.0 A for 30 s, the hetero-interfaces of the Cu/Zn-CP-1-30 electrode showed a porous 3D network structure containing Cu/Zn heterostructures (Fig. 1b). However, smaller deposition currents and shorter deposition times failed to create pore structures. Disordering morphology was also formed either at high deposition current density or long deposition time, resulting in the collapse of the porous 3D network, as shown in Fig. S1 and S2.† We then used the Cu/Zn-CP-1-30 electrode as a representative example for further characterization. Scanning electron microscopy (SEM) images showed that the original CP had a 3D network structure (Fig. 1c). After deposition, the Cu/Zn-CP-1-30 electrode showed typical porous structures containing branched Cu nanodendrites crosslinked with plenty of Zn nanosheets (Fig. 1d-top view and Fig. 1e). The average thickness of Cu/Zn-CP-1-30 layers was about 30 μm (Fig. 1d-side view) and the loading of the active material was approximately 2.5 mg cm^−2^. The transmission electron microscopy (TEM) image of the Cu/Zn-CP-1-30 electrode further indicates the formation of Cu/Zn heterostructures, which is consistent with the SEM result (Fig. 1f). As shown in Fig. 1g, the high-resolution-transmission electron microscopy (HR-TEM) images showed a tight interface between the two components with different lattice stripe structures, indicating the formation of Cu/Zn heterostructures with interfacial boundaries.^28,29^ The interplanar *d*-spacing values were 2.10 Å and 2.33 Å, corresponding to the (111) lattice plane of Cu and the (100) plane of Zn, respectively (Fig. S3†). The EDS mappings further prove the formation of heterostructures, in which the pink regions in the images belong to Cu-containing portions of the nano-dendrites, whereas the yellow regions are the Zn-containing segments (Fig. 1h). For comparison, Cu-CP-1-30 and Zn-CP-1-30 electrodes were also prepared and characterized. SEM demonstrated that they possessed Cu nano-dendrite and Zn nanosheet structures (Fig. S4 and S5†), respectively. The selected area of HR-TEM revealed the single-crystalline structure of each nanograin, with the interplanar spacings of 2.09 and 2.29 Å that corresponded well to the (111) plane of Cu and the (100) plane of Zn (Fig. S6 and S7†), respectively.

**Fig. 2:**
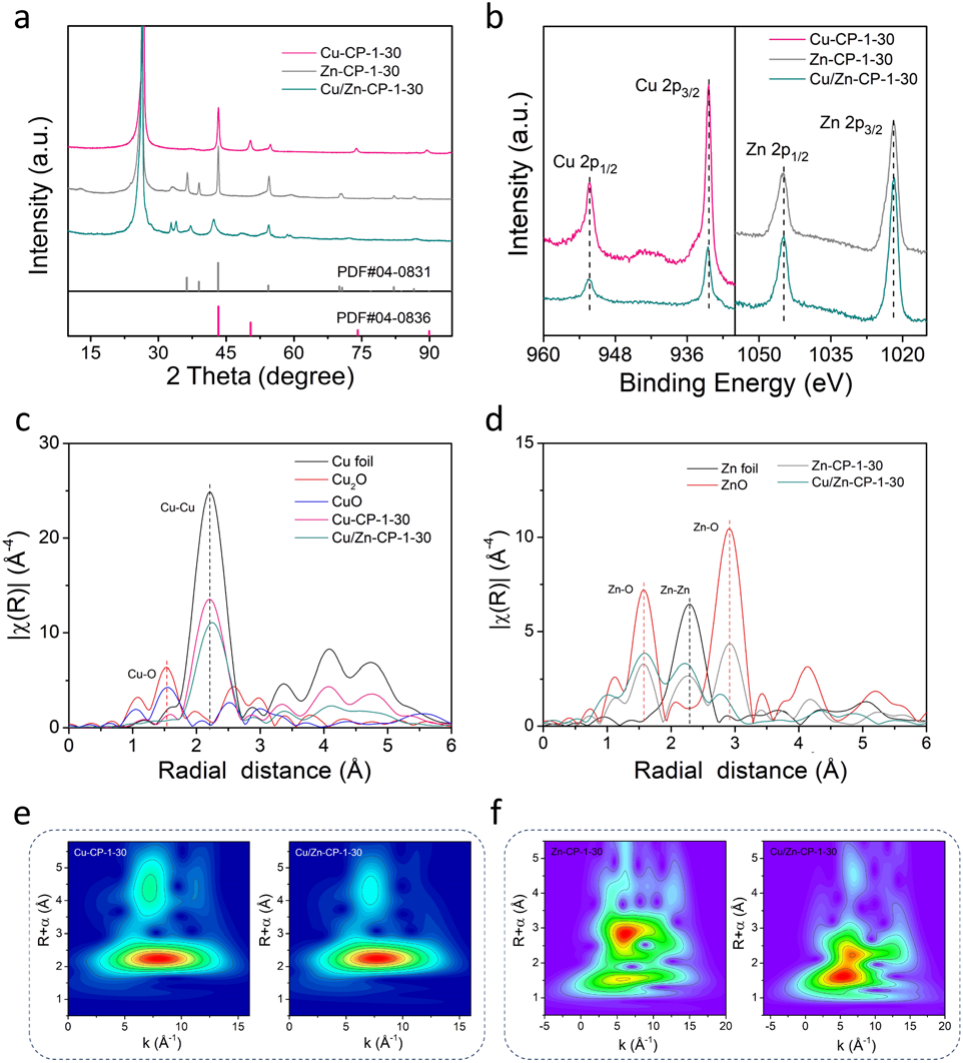
(a) XRD patterns of Cu-CP-1-30, Zn-CP-1-30, and Cu/Zn-CP-1-30 electrodes. (b) XPS spectra of Cu 2p and Zn 2p of different electrodes. Fourier-transformed (FT) *k*^3^-weighted (c) Cu K-edge EXAFS data and (d) Zn K-edge EXAFS data for different electrodes. For comparison, reference spectra from a Cu foil, Zn foil, Cu_2_O, CuO, and ZnO are also shown. Morlet WT of the *k*^3^-weighted EXAFS data of (e) Cu-CP-1-30 and Cu/Zn-CP-1-30 electrodes and (f) Zn-CP-1-30 and Cu/Zn-CP-1-30 electrodes.

The X-ray diffraction (XRD) pattern of the Cu/Zn-CP-1-30 electrode is shown in Fig. 2a. The peaks located at 42.4° and 48.36° can be indexed to the (111) and (200) crystalline planes of Cu, and those at 37.6°, 42.4°, and 54.4° corresponded to the (100), (101), and (102) crystalline planes of Zn. However, Zn-CP-1-30 and Cu-CP-1-30 electrodes only show isolated diffraction peaks of Zn and Cu, respectively. In comparison with Zn-CP-1-30 and Cu-CP-1-30 electrodes, the slightly shifted diffraction peaks of Cu or Zn phases in the Cu/Zn-CP-1-30 electrode also indicate the formation of Cu/Zn heterostructures, which is probably due to lattice distortion near the hetero-interfaces.^30^ This conclusion was further confirmed by the X-ray photoelectron spectroscopy (XPS) analysis, where the binding energies of both Cu 2p and Zn 2p were present in the Cu/Zn-CP-1-30 electrode. In detail, the peaks at 932.4 eV (Cu 2p_3/2_) and 952.3 eV (Cu 2p_1/2_) retained the characteristics of Cu species, while the peaks at 1021.9 eV (Zn 2p_3/2_) and 1044.9 eV (Zn 2p_1/2_) retained the characteristics of Zn species (Fig. 2b). Auger Cu LMM spectra suggest that the element valence states of Cu were Cu^0^ and Cu^+^, and Cu^0^ was predominant (Fig. S8†).^31^ In addition, Cu/Zn heterostructures that were prepared at different current densities and time were also characterized *via* XRD and XPS (Fig. S9 and S10†), and they had similar crystallinity and surface compositions to those of the Cu/Zn-CP-1-30 electrode.

To gain more detailed information about the chemical state and structure of the electrocatalysts, X-ray absorption spectroscopy (XAS) measurements were conducted. Fig. S11† shows selected Cu and Zn K-edge XANES spectra for Cu-CP-1-30, Zn-CP-1-30, and Cu/Zn-CP-1-30 electrodes with reference samples. The extended X-ray absorption fine structure (EXAFS) spectra of the Cu K-edge and Zn K-edge of the as-prepared electrodes are shown in Fig. 2c and d, respectively. As shown in Fig. 2c, the main finding from the Fourier transformed (FT) Cu K-edge EXAFS data analysis is that the Cu–M interatomic distance is different in the Cu/Zn-CP-1-30 electrode as compared to the Cu-CP-1-30 electrode and Cu foil.^32,33^ When comparing the spectra with reference samples, the distinct feature at 2–2.5 Å corresponded to Cu–Cu shells in the Cu/Zn-1-30 electrode, which is similar to what was observed for the Cu foil and Cu-CP-1-30 electrode (Fig. 2e and S12†). Similarly, ZnO, Zn-CP-1-30, and Cu/Zn-CP-1-30 electrodes exhibit a distinct feature at 1.5–2.5 Å, which represents the Zn–Zn shell (Fig. 2f and S13†), whereas a slightly wider WT maximum is observed at 2.5–3 Å, corresponding to Zn–O and Zn–Zn shells. The above analysis indicates that the Cu/Zn heterostructures could create more active sites and facilitate charge transfer between the two metals, leading to a more efficient catalytic process. In addition, Cu/Zn heterostructures have also been proven to exhibit better stability compared to monometallic catalysts. This is due to the strong metal–metal interaction at the interface of the two metals, which prevents the aggregation of active sites and maintains the catalytic activity over a longer period.^34,35^

**Fig. 3:**
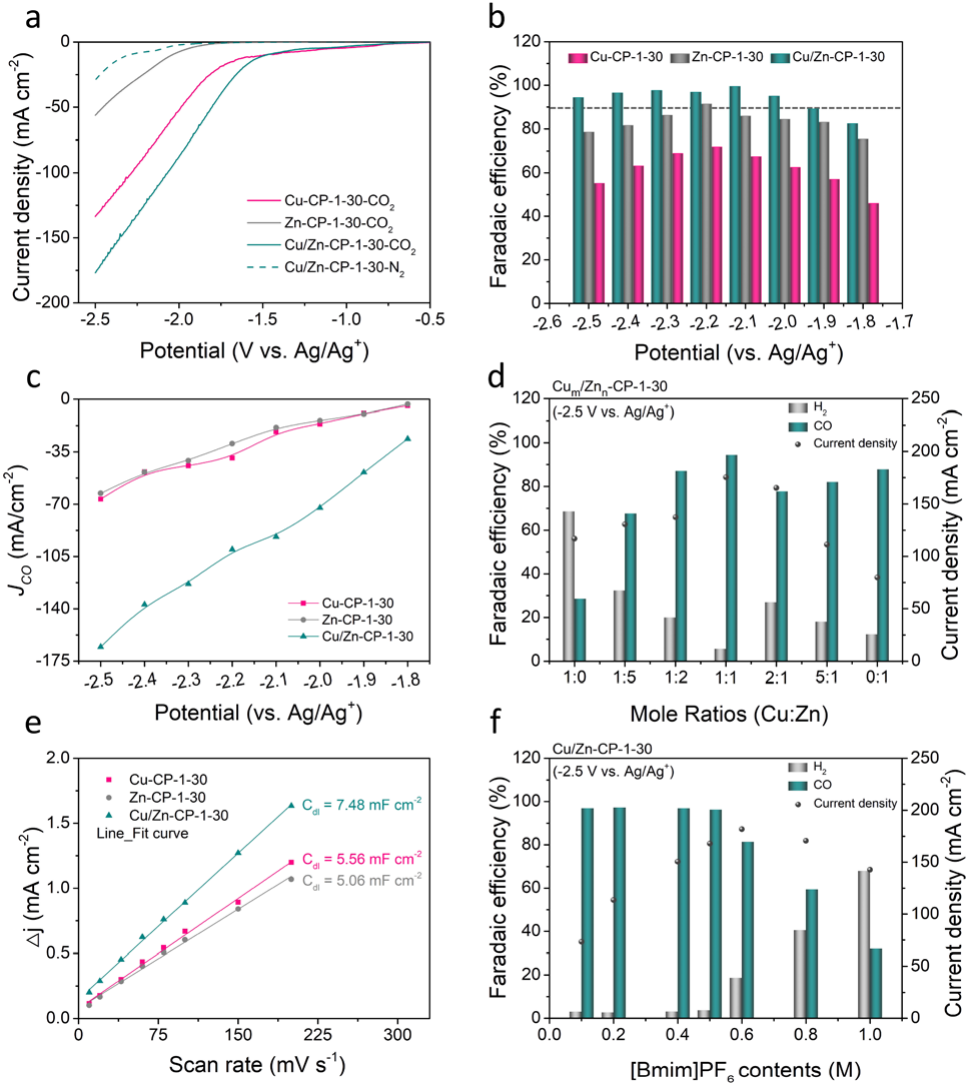
(a) LSV curves of the as-prepared Cu-CP-1-30, Zn-CP-1-30, and Cu/Zn-CP-1-30 electrodes in N_2_ and CO_2_-saturated electrolyte. (b) The FE(CO) over Cu-CP-1-30, Zn-CP-1-30, and Cu/Zn-CP-1-30 electrodes at different applied potentials. (c) The partial current density for CO production for Cu-CP-1-30, Zn-CP-1-30, and Cu/Zn-CP-1-30 electrodes. (d) FE of CO and H_2_ and current density over Cu_*m*_/Zn_*n*_-CP-1-30 electrodes with different Cu : Zn mole ratios using 0.5 M [Bmim]PF_6_/MeCN electrolytes. (e) The measured double-layer capacitance for Cu-CP-1-30, Zn-CP-1-30, and Cu/Zn-CP-1-30 electrodes. (f) FE of CO and H_2_ and current density using electrolytes with different [Bmim]PF_6_ content over Cu/Zn-CP-1-30 electrodes. Electrolysis experiments were carried out at −2.5 V *vs.* Ag/Ag^+^. Data were obtained at ambient temperature and pressure with a CO_2_ stream of 15 sccm.

To assess the electrocatalytic properties of ECR, the as-prepared Cu/Zn-CP-1-30 electrode was directly used as the working electrode in an H-type cell for the electrochemical measurements, in which 0.5 M 1-butyl-3-methylimidazolium hexafluorophosphate ([Bmim]PF_6_)/MeCN was used as the electrolyte. The linear sweep voltammetry (LSV) curves over various electrodes were conducted in a potential range of −0.5 to −2.5 V *vs.* Ag/Ag^+^, and the current density was normalized by geometry area. As shown in Fig. 3a, the Cu/Zn-CP-1-30 electrode displayed superior current density responses and lower onset potential in CO_2_-saturated electrolyte than that in N_2_-saturated electrolyte, indicating the occurrence of ECR. Note that the current density of the Cu/Zn-CP-1-30 electrode was much higher than that of other catalysts in the potential range from −0.5 V to −2.5 V *vs.* Ag/Ag^+^, suggesting ECR was kinetically favorable on the porous Cu/Zn heterostructures. The electrochemical ECR performance of the as-prepared electrodes was also investigated by electrolysis of CO_2_ at different applied potentials ranging from −1.8 V to −2.5 V *vs.* Ag/Ag^+^. The gas products and the liquid products were quantified by gas chromatography (GC) and nuclear magnetic resonance (NMR) spectroscopy, respectively. According to the product distribution, only two products, CO and H_2_, were detected with a total FE of around 100%. As shown in Fig. 3b, the FE of CO was higher than 90% within a wide potential window ranging from −1.9 V to −2.5 V *vs.* Ag/Ag^+^. At −2.1 V *vs.* Ag/Ag^+^, the maximum FE of CO could reach 99.5% with a partial current density of 91.8 mA cm^−2^, which was roughly 4.9 and 4.26 times higher than that over Zn-CP-1-30 and Cu-CP-1-30 electrodes, respectively (Fig. 3c). Comparably, the maximum CO FEs for Zn-CP-1-30 and Cu-CP-1-30 electrodes were only 91.4% (*j*_CO_: −39.1 mA cm^−2^) and 71.8% (*j*_CO_: −29.4 mA cm^−2^), respectively. It is also worth mentioning that over the Cu/Zn-CP-1-30 electrode, the FE of CO could maintain as high as 94.3% at −2.5 V *vs.* Ag/Ag^+^ and the partial current density of CO could reach 165.5 mA cm^−2^. A comparison of representative CO_2_ to CO reduction catalysts revealed that the FE of CO and the partial current density over the Cu/Zn-CP-1-30 electrode were much higher than most reported electrocatalysts in an H-type cell (Table S1†).

Considering the experimental observations above, we think that the Cu/Zn-CP-1-30 electrode can inhibit the production of H_2_ and improve the selectivity of CO *via* the formation of porous Cu/Zn hetero-interfaces. Apparently, the Cu/Zn heterostructures prepared at different deposition currents and deposition times affect the catalytic activity of ECR for CO. As shown in Fig. S14–S17,† in comparison with the Cu/Zn-CP-1-30 electrode, lower selectivity of CO was obtained over Cu/Zn heterostructures that were prepared at either low deposition current density or longer deposition time. This indicates that the preparing conditions influence the physical features of Cu/Zn heterostructures (see Fig. 1b, S1 and S2†) such as the porous surface structure and the formation of heterostructures, which in turn influence mass/ion transportation and ECR kinetics. To prove this, we prepared different porous Cu/Zn heterostructures with different mole ratios of Cu_*m*_/Zn_*n*_ according to their distinct diffraction intensities, and the contents of Cu/Zn mole ratios determined by inductively coupled plasma optical emission spectroscopy (ICP-OES) are illustrated in Table S2.† Their FEs and *j*_CO_ values strongly depend on the Cu/Zn heterostructures with different mole ratios (Fig. 3d). In addition, the electrochemical active surface area (ECSA) was then estimated through the electrochemical double-layer capacitance (*C*_dl_) measurements. The cyclic voltammetry (CV) curves with various scan rates at the non-faradaic region were recorded in Fig. S18† for all the catalysts. The linear slopes in Fig. 3e show that the *C*_dl_ value for the Cu/Zn-CP-1-30 electrode was higher than that of the Zn-CP-1-30 electrode and Cu-CP-1-30 electrode, indicating that the Cu/Zn-CP-1-30 electrode could afford more active sites in ECR.^36^

It is also well known that ionic liquids play an import role as electrolyte in ECR. The presence of ionic liquids can greatly improve the solubility of CO_2_ and ensure the effective supply of CO_2_ in the ECR process, thus inhibiting the hydrogen evolution reaction (HER) and improving the catalytic activity and selectivity of ECR.^37–41^ Therefore, we tested the ECR catalytic performance over the Cu/Zn-CP-1-30 electrode using electrolytes with different concentrations of [Bmim]PF_6_/MeCN at −2.5 V *vs.* Ag/Ag^+^. It can be observed that the FE of CO changed with [Bmim]PF_6_ content in the electrolytes (Fig. 3f). When the electrolyte contained 0.5 M [Bmim]PF_6_, it exhibited optimal FE of CO and current density, which was superior to other [Bmim]PF_6_ contents, indicating that the contents of [Bmim]PF_6_ play an essential role in the reaction.^10^ The long-term electrolysis stability of the catalyst is also an essential factor for the viable application for ECR. We then investigated the electrode stability of the Cu/Zn-CP-1-30 electrode at −2.1 V *vs.* Ag/Ag^+^ for 16 h. As shown in Fig. S19,† both the current density and the FE of CO remained stable during the entire electrolysis period. As demonstrated in SEM (Fig. S20†) and XAFS (Fig. S21 and S22†), no noticeable change in morphology, crystallographic structure, and composition was observed over the Cu/Zn-CP-1-30 electrode, suggesting that pore structure and the physical phase keep stable during the ECR reaction.

**Fig. 4:**
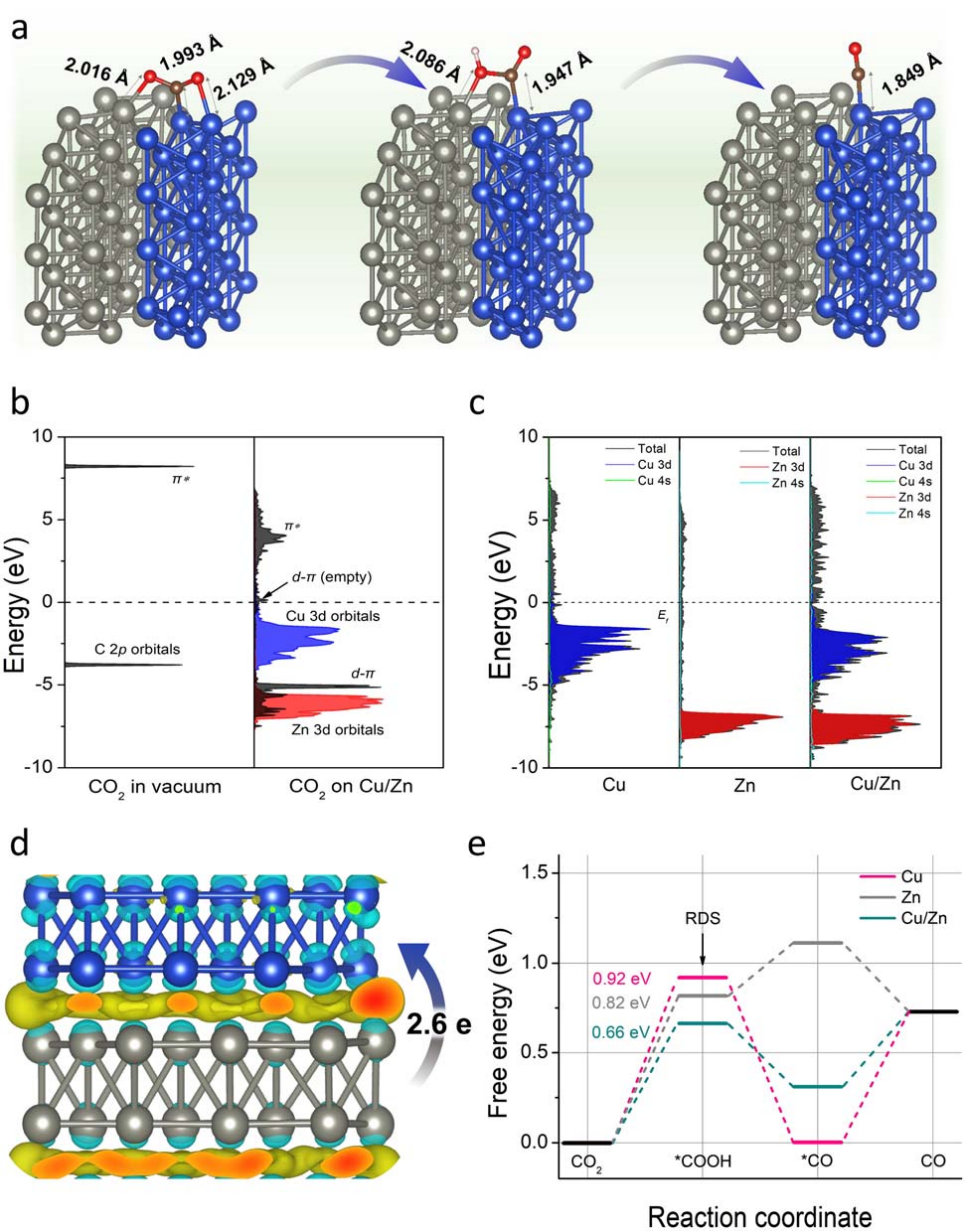
(a) The adsorption state of CO_2_, *COOH, and *CO on the Cu/Zn-1-30 electrode. The blue, gray, brown, red, and white balls represent Cu, Zn, C, O, and H atoms, respectively. (b) Projected electronic densities of states of the C 2p orbitals of CO_2_ in a vacuum, and the Cu 3d/Zn 3d orbitals and the C 2p orbitals of CO_2_ adsorbed on Cu/Zn heterostructures. (c) Densities of states of Cu-CP, Zn-CP, and Cu/Zn-CP heterostructures. (d) Top view of the charge density difference of Cu/Zn hetero-interfaces with an isosurface of 3.6 × 10^−3^ e Å^−3^. (The charge accumulation is shown as the yellow region, and the charge depletion is shown as the cyan region.) (e) Gibbs free-energy diagrams for ECR to CO over the simulated Cu/Zn-CP-1-30 electrode.

To further verify the above conclusion, we conducted density functional theory (DFT) calculations to gain insight into the working mechanism of Cu/Zn heterostructures. As depicted in computational structure models in Fig. S23,† the models of Cu(111), Zn(100), and Cu(111)/Zn(100) were represented by Cu-CP-1-30, Zn-CP-1-30, and Cu/Zn-CP-1-30 electrodes, respectively, and the interactions and electronic structure among Cu and Zn atoms are different for the catalysts. In a CO_2_-saturated solution, the negatively charged sites in Cu/Zn heterostructures can capture a CO_2_ molecule, *COOH and *CO easily. As shown in Fig. 4a, the C–Cu bond lengths from CO_2_, *COOH, and *CO are gradually reduced to 1.993 Å, 1.947 Å, and 1.849 Å over Cu/Zn heterogeneous sites, indicating strong chemical adsorption of the intermediates. The chemical adsorption of CO_2_ is also confirmed by the charge transfer from the Cu/Zn heterogeneous sites to CO_2_ molecules. We first investigated the projected densities of states of CO_2_ adsorption. As shown in Fig. 4b and c, the Zn 3d orbitals of Cu/Zn overlap with the π orbitals of CO_2_ and their energies are lower than the π* orbitals, indicating that the adsorbate state is dominated by d–π interactions.^42^ When coupling the Cu 3d orbitals and Zn 3d orbitals in Cu/Zn, the π orbitals are broadened and split into d–π bonding and anti-bonding orbitals below and above the original energy level. The narrower d-band center (εd) of Cu/Zn heterostructures compared to Cu(111) and Zn(100) contributes to the formation of d–π antibonding orbitals above the Fermi level (εF).^43,44^ The additional formation of empty orbitals promotes the transfer of electrons and indicates a further decrease in the activation energy for CO formation on Cu/Zn heterostructures.^45,46^ The charge density differences of the Cu/Zn heterostructures are given in Fig. 4d and S24.† It shows that electron-deficient and electron-rich regions appeared around the Cu and Zn atoms, respectively, indicating that there is a significant charge redistribution at the Cu/Zn hetero-interfaces, and the surface transfer of electrons from Zn to Cu can increase the electron density at the Cu/Zn hetero-interfaces and enhance the electron transport properties at the interface. The surface transfer of electrons from Zn to Cu may help to improve the catalytic activity and selectivity at the Cu/Zn hetero-interfaces, thus enhancing the electrocatalytic performance of ECR.^47–49^

To further elucidate the mechanism of the excellent ECR performance on the Cu/Zn-CP-1-30 electrode, we also calculated the energy barriers for the whole reaction pathway for ECR. As shown in Fig. 4e, it is clear that the Cu-CP-1-30 electrode exhibits the highest energy barrier (0.92 eV) at the first hydrogenation step, and this energy barrier is lowered to 0.82 eV on the Zn-CP-1-30 electrode. In particular, the Cu/Zn-CP-1-30 electrode reduces the energy barrier to 0.66 eV, because the heterostructure sites can improve the adsorption of the *COOH intermediate and facilitate the formation of CO.^50^ Taken together, our control experiments and DFT calculations provide compelling evidence certifying that the porous and self-supported architecture is crucial for fast mass/ion diffusion and the Cu/Zn heterostructures can produce an interfacial bonding effect, offering more active sites for ECR to CO.

## Conclusions

In summary, we find that ultra-fast synthesized 3D porous Cu/Zn heterostructures *via in situ* electrodeposition possessed a high activity and selectivity for the ECR to CO. Over the Cu/Zn-CP-1-30 electrode, the FE of CO could reach nearly 100% with a very high partial current density of 91.8 mA cm^−2^ at −2.1 V *vs.* Ag/Ag^+^. In particular, the partial current density of CO could reach 165.5 mA cm^−2^ and the FE of CO could remain as high as 94.3% at −2.5 V *vs.* Ag/Ag^+^ in an H-type cell. The outstanding electrocatalytic performance of the electrode can be ascribed to the formation of 3D porous Cu/Zn heterostructures, in which the porous and self-supported architecture facilitates fast mass/ion diffusion and the Cu/Zn heterostructures promote the adsorption of the *COOH intermediate in the process of CO formation. In addition, the electrode was very stable. We believe that the efficient electrode has promising potential for application in ECR. The ultra-fast and simple strategy for synthesizing 3D porous heterostructures can also be used to design other efficient electrodes for ECR.

## Data availability

The authors declare that all data supporting the findings of this study are available within the paper [and its ESI†].

## Author contributions

S. Q. J., Q. G. Z., H. H. W. and B. X. H. proposed the project, designed the experiments, and wrote the manuscript. S. Q. J. performed all the experiments. S. T. H., J. X. Z., M. K. D., W. X. and X. Q. X. performed the analysis of experimental data. Q. G. Z., H. H. W., M. Y. H. and B. X. H. co-supervised the whole project. All authors discussed the results and commented on the manuscript.

## Conflicts of interest

The authors declare no competing interests.

## Supplementary Material

SC-014-D3SC03317A-s001

## References

[cit1] Gao P., Li S., Bu X., Dang S., Liu Z., Wang H., Zhong L., Qiu M., Yang C., Cai J., Wei W., Sun Y. (2017). Nat. Chem..

[cit2] Li F., Li Y. C., Wang Z., Li J., Nam D.-H., Lum Y., Luo M., Wang X., Ozden A., Hung S.-F., Chen B., Wang Y., Wicks J., Xu Y., Li Y., Gabardo C. M., Dinh C.-T., Wang Y., Zhuang T.-T., Sinton D., Sargent E. H. (2020). Nat. Catal..

[cit3] Wei J., Ge Q., Yao R., Wen Z., Fang C., Guo L., Xu H., Sun J. (2017). Nat. Commun..

[cit4] Yang D., Zhu Q., Han B. (2020). Innovation.

[cit5] Jin S., Hao Z., Zhang K., Yan Z., Chen J. (2021). Angew. Chem., Int. Ed..

[cit6] Ma M., Zheng Z., Yan W., Hu C., Seger B. (2022). ACS Energy Lett..

[cit7] Teh W. J., Kolb M. J., Calle-Vallejo F., Yeo B. S. (2023). Adv. Funct. Mater..

[cit8] Kang M. P. L., Kolb M. J., Calle-Vallejo F., Yeo B. S. (2022). Adv. Funct. Mater..

[cit9] Hori Y., Wakebe H., Tsukamoto T., Koga O. (1994). Electrochim. Acta.

[cit10] Rosen B. A., Salehi-Khojin A., Thorson M. R., Zhu W., Whipple D. T., Kenis P. J. A., Masel R. I. (2011). Science.

[cit11] Jin S., Hao Z., Zhang K., Yan Z., Chen J. (2021). Angew. Chem., Int. Ed..

[cit12] Xiao J., Gao M.-R., Liu S., Luo J.-L. (2020). ACS Appl. Mater. Interfaces.

[cit13] Yang D., Zhu Q., Sun X., Chen C., Guo W., Yang G., Han B. (2020). Angew. Chem., Int. Ed..

[cit14] Wu Y., Chen C., Yan X., Sun X., Zhu Q., Li P., Li Y., Liu S., Ma J., Huang Y., Han B. (2021). Angew. Chem., Int. Ed..

[cit15] Yin Y. Y., Kang X. C., Han B. X. (2022). Chem. Synth..

[cit16] Fan Q., Zhang X., Ge X., Bai L., He D., Qu Y., Kong C., Bi J., Ding D., Cao Y., Duan X., Wang J., Yang J., Wu Y. (2021). Adv. Energy Mater..

[cit17] Fu H. Q., Liu J., Bedford N. M., Wang Y., Sun J. W., Zou Y., Dong M., Wright J., Diao H., Liu P., Yang H. G., Zhao H. (2022). Adv. Mater..

[cit18] Meng Y. C., Kuang S. Y., Liu H., Fan Q., Ma X. B., Zhang S. (2021). Acta Phys.–Chim. Sin..

[cit19] Xing Y., Kong X., Guo X., Liu Y., Li Q., Zhang Y., Sheng Y., Yang X., Geng Z., Zeng J. (2020). Adv. Sci..

[cit20] Liang Y., Zhou W., Shi Y., Liu C., Zhang B. (2020). Sci. Bull..

[cit21] Zhang J., Sun W., Ding L., Wu Z., Gao F. (2021). ACS Sustainable Chem. Eng..

[cit22] Wu Q., Mao M.-J., Wu Q.-J., Liang J., Huang Y.-B., Cao R. (2021). Small.

[cit23] Yuan X., Wu Y., Jiang B., Wu Z., Tao Z., Lu X., Liu J., Qian T., Lin H., Zhang Q. (2020). ACS Appl. Mater. Interfaces.

[cit24] Zhang Z., Wen G., Luo D., Ren B., Zhu Y., Gao R., Dou H., Sun G., Feng M., Bai Z., Yu A., Chen Z. (2021). J. Am. Chem. Soc..

[cit25] Ozden A., Wang Y., Li F., Luo M., Sisler J., Thevenon A., Rosas-Hernández A., Burdyny T., Lum Y., Yadegari H., Agapie T., Peters J. C., Sargent E. H., Sinton D. (2021). Joule.

[cit26] Gao J., Zhang H., Guo X., Luo J., Zakeeruddin S. M., Ren D., Gratzel M. (2019). J. Am. Chem. Soc..

[cit27] Plowman B. J., Jones L. A., Bhargava S. K. (2015). Chem. Commun..

[cit28] Zhao Y., Zhang X.-G., Bodappa N., Yang W.-M., Liang Q., Radjenovica P. M., Wang Y.-H., Zhang Y.-J., Dong J.-C., Tian Z.-Q., Li J.-F. (2022). Energy Environ. Sci..

[cit29] Zhang Y., Han X., Liu R., Yang Z., Zhang S., Zhang Y., Wang H., Cao Y., Chen A., Sun J. (2022). Small.

[cit30] Su J., Li G.-D., Li X.-H., Chen J.-S. (2019). Adv. Sci..

[cit31] Belgamwar R., Verma R., Das T., Chakraborty S., Sarawade P., Polshettiwar V. (2023). J. Am. Chem. Soc..

[cit32] Gao J., Zhang H., Guo X., Luo J., Zakeeruddin S. M., Ren D., Gratzel M. (2019). J. Am. Chem. Soc..

[cit33] Zhao Y., Liu X., Chen D., Liu Z., Yang Q., Lin X., Peng M., Liu P., Tan Y. (2021). Sci. China Mater..

[cit34] Shi R., Guo J., Zhang X., Waterhouse G. I. N., Han Z., Zhao Y., Shang L., Zhou C., Jiang L., Zhang T. (2020). Nat. Commun..

[cit35] Shen H., Zhao Y., Zhang L., He Y., Yang S., Wang T., Cao Y., Guo Y., Zhang Q., Zhang H. (2023). Adv. Energy Mater..

[cit36] Rahaman M., Dutta A., Zanetti A., Broekmann P. (2017). ACS Catal..

[cit37] Rosen B. A., Salehi-Khojin A., Thorson M. R., Zhu W., Whipple D. T., Kenis P. J. A., Masel R. I. (2011). Science.

[cit38] Zhu Q., Ma J., Kang X., Sun X., Liu H., Hu J., Liu Z., Han B. (2016). Angew. Chem., Int. Ed..

[cit39] Yang J., Kang X., Jiao J., Xing X., Yin Y., Jia S., Chu M., Han S., Xia W., Wu H., He M., Han B. (2023). J. Am. Chem. Soc..

[cit40] Sun X., Lu L., Zhu Q., Wu C., Yang D., Chen C., Han B. (2018). Angew. Chem., Int. Ed..

[cit41] Li P., Bi J., Liu J., Zhu Q., Chen C., Sun X., Zhang J., Han B. (2022). Nat. Commun..

[cit42] Zhou Y., Sun W., Chu W., Zheng J., Gao X., Zhou X., Xue Y. (2018). Appl. Surf. Sci..

[cit43] Bligaard T., Nørskov J. K. (2007). Electrochim. Acta.

[cit44] Kim D., Resasco J., Yu Y., Asiri A. M., Yang P. (2014). Nat. Commun..

[cit45] Wang J., Zheng X., Wang G., Cao Y., Ding W., Zhang J., Wu H., Ding J., Hu H., Han X., Ma T., Deng Y., Hu W. (2022). Adv. Mater..

[cit46] Lee J. H., Kattel S., Jiang Z., Xie Z., Yao S., Tackett B. M., Xu W., Marinkovic N. S., Chen J. G. (2019). Nat. Commun..

[cit47] Sultan S., Lee H., Park S., Kim M. M., Yoon A., Choi H., Kong T.-H., Koe Y.-J., Oh H.-S., Lee Z., Kim H., Kim W., Kwon Y. (2022). Energy Environ. Sci..

[cit48] Clark E. L., Hahn C., Jaramillo T. F., Bell A. T. (2017). J. Am. Chem. Soc..

[cit49] Ma S., Sadakiyo M., Heima M., Luo R., Haasch R. T., Gold J. I., Yamauchi M., Kenis P. J. A. (2017). J. Am. Chem. Soc..

[cit50] Yin J., Jin J., Yin Z., Zhu L., Du X., Peng Y., Xi P., Yan C.-H., Sun S. (2023). Nat. Commun..

