# Supplementary material for: Ultra-fast synthesis of three-dimensional porous Cu/Zn heterostructures for enhanced carbon dioxide electroreduction
Source: Chem Sci. 2023 Oct 4;14(41):11474–80. doi: 10.1039/d3sc03317a (PMC10599477; doi:10.1039/d3sc03317a)
Supplement: SC-014-D3SC03317A-s001 [file SC-014-D3SC03317A-s001.pdf]

Electronic Supplementary Material (ESI) for Chemical Science.
This journal is © The Royal Society of Chemistry 2023

Supporting Information

Ultra-fast synthesis of three-dimensional Porous Cu/Zn Heterostructures for Enhanced Carbon Dioxide Electroreduction

Shuaiqiang Jia,[a] Qinggong Zhu,*[b, c] Shitao Han,[a] Jianxin Zhai,[a] Mengke Dong,[a] Wei Xia,[a] Xueqing Xing,[d] Haihong Wu,*[a, e] Mingyuan He,[a, e] and Buxing Han*[a, b, c, e]

[a] Shanghai Key Laboratory of Green Chemistry and Chemical Processes, School of Chemistry and Molecular Engineering, East China Normal University, Shanghai, 200062 (China)

[b] Beijing National Laboratory for Molecular Sciences, Key Laboratory of Colloid and Interface and Thermodynamics, Center for Carbon Neutral Chemistry, Institute of Chemistry, Chinese Academy of Sciences, Beijing, 100190 (China)

[c] School of Chemistry, University of Chinese Academy of Sciences, Beijing, 100049 (China)

[d] Beijing Synchrotron Radiation Facility, Institute of High Energy Physics, Chinese Academy of Sciences, Beijing, 100049 (China)

[e] Institute of Eco-Chongming, 20 Cuiniao Road, Chenjia Town, Chongming District, Shanghai, 202162 (China)

Content:

Experimental Section……………….………………………..……………………….…...………....3

Materials……………….……….................…………………….…………..…………….…………....3

Catalyst Preparation…………….....…......……………..……………………………….…...………......3

Characterization…………….……….....……………….…………………….…………………...……....4

Electrochemical Study………........……………….…………….……….……...……………..….…………...4

Products Analysis…………………….……………………….……………………….……...…………....5

Determined of Double-layer Capacitances (C_{dl}) Measurement……………………...…………......5

Computational Method…………………….……………………….……….………….………….……..5

Supplementary Figures and Tables…………………….…………………….…….……………...…7

References…………………………….………………………….…………………….……...……….........31

Experimental Section

Materials

Copper (II) sulfate pentahydrate ($CuSO_4 \cdot 5H_2O$), zinc (II) sulfate heptahydrate ($ZnSO_4 \cdot 7H_2O$), ammonium sulfate ($(NH_4)_2SO_4$), acetonitrile (CH_3CN) and sulfuric acid (H_2SO_4) were obtained from Sinopharm Chemical Reagent Co., Ltd. [Bmim]PF_6 (purity>99%) was purchased from Lanzhou Yulu Fine Chemical Co., Ltd. Copper (Cu) standard solution, and zinc (Zn) standard solution were provided by Shanghai Aladdin Bio-Chem Technology Co., LTD. Toray Carbon Paper (CP, TGP-H-60, 19×19 cm), Nafion N-117 membrane (0.180 mm thick, ≥0.90 meg/g exchange capacity) and Nafion D-521 dispersion (5% w/w in water and 1-propanol, ≥ 0.92 meg/g exchange capacity) were purchased from Alfa Aesar China Co., Ltd. Both CO_2 and N_2 had a purity of 99.999 %, which were provided by Shanghai Chemistry Industrial Zone Pujiang Special Type Gas Co., Ltd. All the chemical reagents were used directly without further purification.

Catalyst Preparation

Synthesis of 3D porous Cu/Zn heterostructures: Porous Cu/Zn heterostructures were deposited on a piece of carbon paper (CP) with a geometric area of 1 cm^2. Before deposition, the CPs were ultrasonically cleaned with acetone, ethanol, and deionized water. The cell was a single-compartment cell, equipped with platinum gauze as the counter electrode. Solutions were prepared from deionized water and high-purity chemicals. For the 3D porous Cu/Zn heterostructures, the electrodeposition was carried out cathodically using the mixed solution of 50 mL H_2SO_4 (20 mM) solution of $CuSO_4$ (50 mM), $ZnSO_4$ (50 mM), and $(NH_4)_2SO_4$ (1.5 M). Electrodepositions were performed at room temperature with different [Cu^{2+}]/[Zn^{2+}] ratios and the total concentration of metal ions was 100 mM. Galvanostatic control was imposed by a DC Power supply (Hangzhou Huayi Electronics Industry Co., Ltd.).

Porous Cu/Zn heterostructures were deposited at a current density of −1.0 A for 30 s. (Cu_m/Zn_n-CP-*x*-*y*; *m/n*: [Cu^{2+}]/[Zn^{2+}] mole ratios, *x*: electrodeposition current density (A cm^{-2}), *y*: electrodeposition time (s)). All data of current density and material loading expressed per unitary area are referred to the geometric surface. The gravimetric data were measured after removing loose deposits around the CP boundary with a moderate water jet and proper drying. The as-prepared electrode was washed with water several times and dried at room temperature in a vacuum oven before use.

Synthesis of Cu-CP or Zn-CP electrodes: For Cu-CP or Zn-CP electrodes, the mixed deposition solution contains only $CuSO_4$ or $ZnSO_4$ metal salts. Any other procedures are the same as above.

Characterization

X-ray diffraction patterns were acquired by an X-ray diffractometer (XRD; Rigaku Ultima VI X-ray) with Cu-Kα radiation (λ=1.54 Å). The morphologies of the samples were observed by field-emission scanning electron microscopy (SEM) (Hitachi S4800) and transmission electron microscope equipped with EDS (TEM, JEM-2100F) operated at 200 kV. The valence states and composition of the samples were examined by X-ray photoelectron spectroscopy (XPS) on an AXIS Supra surface analysis instrument using a monochromatic Al Kα X-ray beam (1,486.6 eV). Before the XPS measurements, the catalysts were stored under ambient conditions. This effectively preserved oxidation of the samples during the sample transfer. The metal content in the catalysts was determined by inductively coupled plasma optical emission spectroscopy (ICP-OES, Optima 8300, Perkin-Elmer). Comparison to Cu and Zn standards of known concentration allowed the determination of the respective Cu and Zn concentrations. The X-ray absorption spectroscopy (XAFS) experiments were carried out at the 4B9A beamline at Beijing Synchrotron Radiation Facility (BSRF), China. Data analysis of Cu K-edge X-ray absorption near-edge spectroscopy (XANES) and extended X-ray absorption fine structure (EXAFS) spectra was conducted using the Athena software package. Pre-edge and post-edge backgrounds were subtracted from the XAS spectra, and the resulting spectra were normalized by edge height.

Electrochemical study

All CO_2 reduction experiments were performed in a gas-tight two-compartment H-cell separated by a proton exchange membrane (Nafion117) using a CHI 660E potentiostat workstation (Shanghai CH Instruments Co., China) at room temperature. The anode and cathode sides were filled with 30 mL of 0.5 M [Bmim]PF_6/MeCN and 0.5 M H_2SO_4, respectively. Before each set experiment, the catholyte was bubbled with CO_2 or N_2 for at least 30 min to form a CO_2-saturated or N_2-saturated solution. Linear sweep voltammetric (LSV) scans were conducted in an H-type cell with a three electrodes configuration, which consisted of a working electrode, a platinum gauze as a counter electrode, and Ag/Ag^+ (Ag/Ag^+ with 0.01 M $AgNO_3$ in 0.1 M TBAP/MeCN solution) as a reference electrode. LSV measurements in gas-saturated electrolytes were carried out in the potential range of –0.5 V to –2.5 V vs. Ag/Ag^+ at a scan rate of 20 mV s^{-1}.

The as-synthesized electrodes were used as the working electrode. The Nafion-117 membrane was used as a proton exchange membrane to separate the cathode and anode compartments in the experiments. In the electrolysis experiment, the amount of electrolyte was 30 mL. The catholyte was bubbled with CO_2 for at least 30 min to form CO_2 saturated solution and the potentiostatic electrochemical reduction was carried out under a steady stream of CO_2 (15 sccm). First, the cathode side was electrochemically reduced using the cyclic voltammetric (CV) method, which ranged from

−0.5 to −2.5 V vs. Ag/Ag^+ at a rate of 0.1 V s^{-1} for 5 cycles to completely reduce the possible oxidized species.

Products analysis

After electrolysis, the gaseous products were collected and analyzed by gas chromatography (GC, Agilent–8890). From the GC peak areas and calibration curves of the TCD detector, the moles of a gaseous product can be calculated. The liquid products were quantified by a nuclear magnetic resonance (NMR) spectrometer. 1H NMR spectra of freshly acquired samples were collected on an NMR spectrometer (Bruker; Ascend 400–400 MHz) in deuterated water (D_2O) with phenol as an internal standard. The mole of a liquid product was calculated from integral areas and calibration curves.

After the quantification, the FE of each product was calculated as follows:

$$FE = (n \times F \times \text{moles of product}) / Q \times 100\%$$

(F: The Faraday constant (96485 C mol^{-1}); *n*: the number of electrons transferred for product formation; Q: the amount of charge passed through the working electrode.)

Determined of double-layer capacitances (C_{dl}) measurement

The value of C_{dl} is in proportion to the electrochemically active surface area. The value of C_{dl} was determined by measuring the capacitive current associated with double-layer charging from the scan-rate dependence of cyclic voltammogram (CV) in an H-type cell. The CV was obtained from −1.5 V to −1.6 V vs. Ag/Ag^+. Scans were recorded at different scan rates with a minimum of 3 cycles in the non-Faradaic region, which included 10 mV s^{-1}, 20 mV s^{-1}, 40 mV s^{-1}, 60 mV s^{-1}, 80 mV s^{-1}, 100 mV s^{-1}, 150 mV $s^{-1,}$ and 200 mV s^{-1}. The C_{dl} was estimated by plotting the Δj ($j_a - j_c$) at –1.55 V vs. Ag/Ag^+ (0.5 M [Bmim]PF_6/MeCN solution) against the scan rates, where j_a and j_c were the anodic and cathodic current density, respectively.

The ECSA of the working electrodes can be calculated according to the following equation: ECSA = R_fS, where R_f is the roughness factor and S is the actual surface area of the working electrode (in this work, S = 1 cm^2). The R_f can be calculated by the relation $R_f = C_{dl}/a$, where a is the double-layer capacitance of a smooth Cu surface. Therefore, the ECSA is proportional to C_{dl} value and can be compared via C_{dl} value.

Computational Method

DFT calculations were conducted through the Vienna ab initio Simulation Package[1-2] (VASP) with the Projector Augment Wave[3] (PAW) method. Generalized gradient approximation of the Perdew-Burke-Ernzerhof (PBE) functional was used as the exchange-correlation functional.[4-5]

The cutoff energy was set as 500 eV, and structure relaxation was performed until the

convergence criteria of energy and force reached 1×10^{-5} eV and 0.02 eV $Å^{-1}$, respectively. The Brillouin zone was sampled with $2 \times 2 \times 1$ K points for surface calculation. A vacuum layer of 15 Å was constructed to eliminate interactions between periodic structures of surface models. The van der Waals (vdW) interaction was amended by the zero damping DFT-D3 method of Grimme.

In aqueous conditions, the reduction of CO_2 to produce CO could occur in the following three elementary steps:

$$CO_2 + (H+ + e^-) + * \rightarrow *COOH$$

$$*COOH + (H^+ + e^-) \rightarrow *CO + H_2O$$

$$*CO \rightarrow CO\uparrow + *$$

where * denotes the active sites on the catalyst surface. Based on the above mechanism, the free energy of two intermediate states, *COOH and *CO, is important to identify the activity of the catalysts. The computational hydrogen electrode (CHE) model[6] proposed by Norskov et al. was used to calculate the free energies of CO_2 reduction intermediates, based on which the free energy of an adsorbed species is defined as:

$$\Delta G = \Delta E + \Delta E_{ZPE} - T\Delta S$$

Where the ΔE, ΔE_{ZPE}, and ΔS are electronic energy, zero-point energy, and entropy difference between products and reactants, respectively. The zero-point energies of isolated and adsorbed intermediates were calculated from the frequency analysis. The vibrational frequencies and entropies of molecules in the gas phase were obtained from the National Institute of Standards and Technology (NIST) database.

Supplementary Figures and Tables

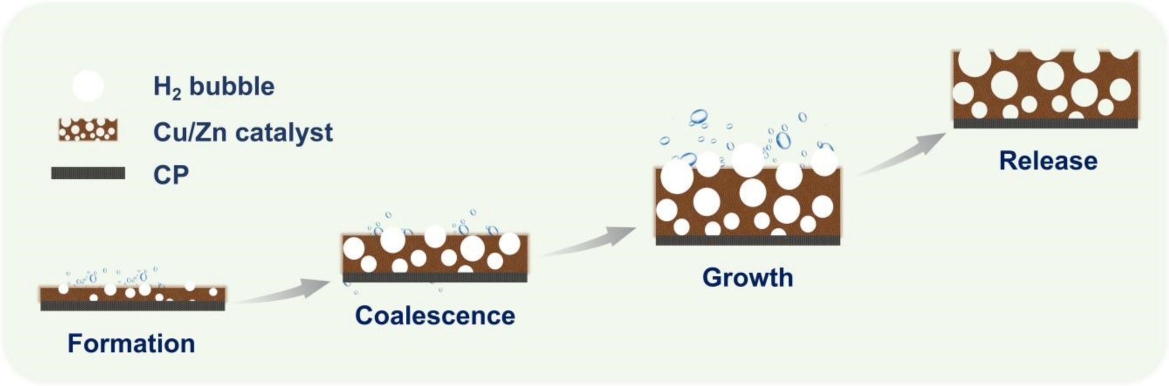

Scheme S1. Schematics of the synthesis of the 3D porous Cu/Zn-CP heterostructures via the dynamic hydrogen bubble template method via electrodeposition.

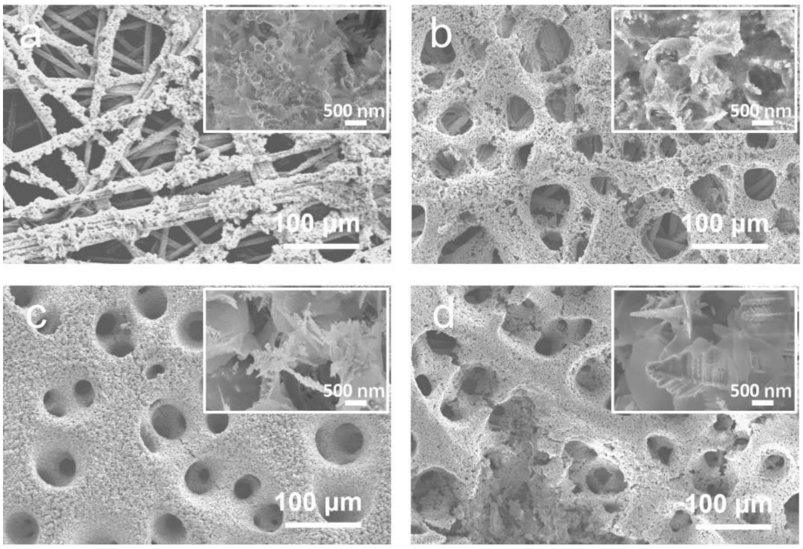

Figure S1. SEM images of (a) Cu/Zn-CP-0.1-30, (b) Cu/Zn-CP-0.5-30, (c) Cu/Zn-CP-1-30, and (d) Cu/Zn-CP-2-30 electrodes with different deposition current densities of −0.1, −0.5, −1.0, and −2.0 A cm^{-2} for 30 s.

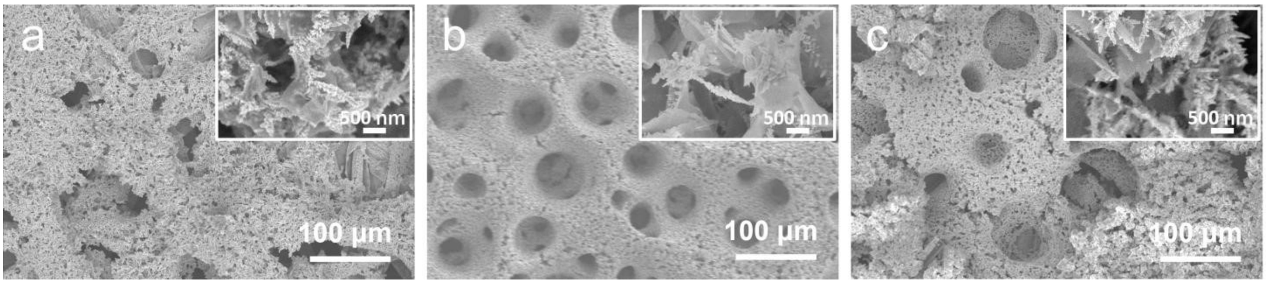

Figure S2. SEM images of (a) Cu/Zn-CP-1-15, (b) Cu/Zn-CP-1-30, and (c) Cu/Zn-CP-1-60 electrodes with a deposition current density of −1.0 A cm^{-2} at different deposition times of 15, 30, and 60 s.

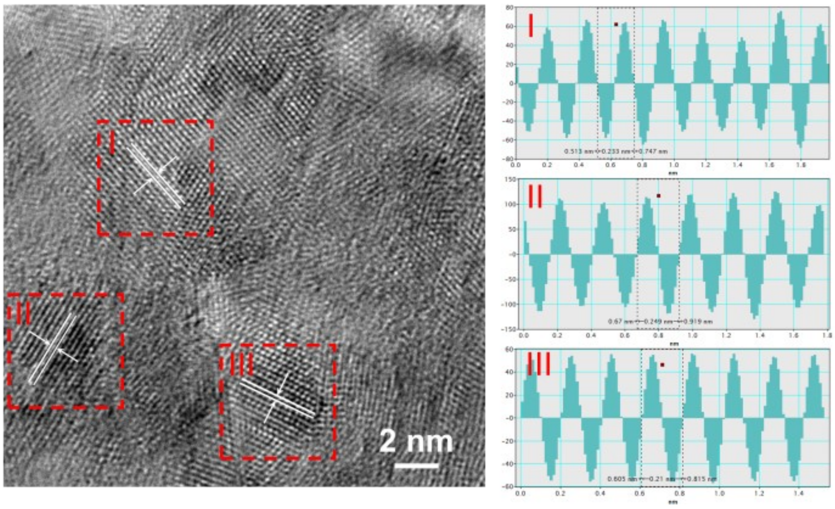

Figure S3. HR-TEM image and intensity profiles measured from the marked area in left HR-TEM image of Cu/Zn-CP-1-30 electrode.

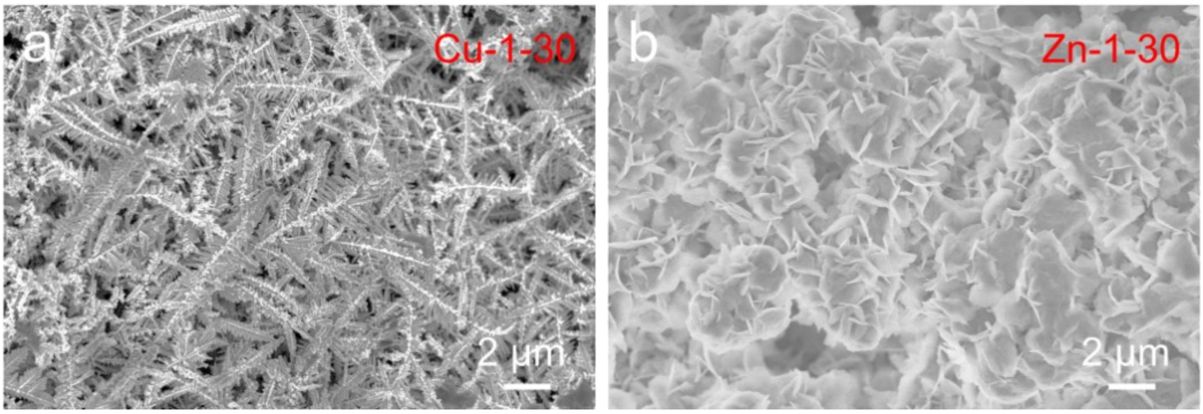

Figure S4. (a) SEM images of Cu-CP-1-30 and (b) Zn-CP-1-30 electrodes with a deposition current density of −1 A cm^{-2} for 30 s.

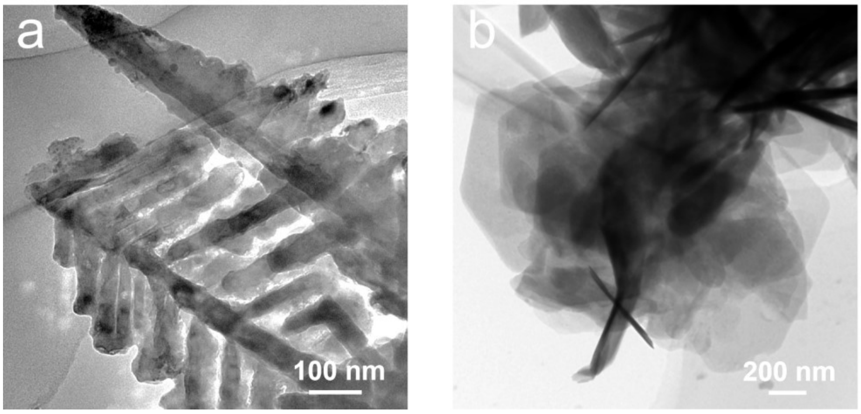

Figure S5. TEM images of (a) Cu-CP-1-30 and (b) Zn-CP-1-30 electrodes.

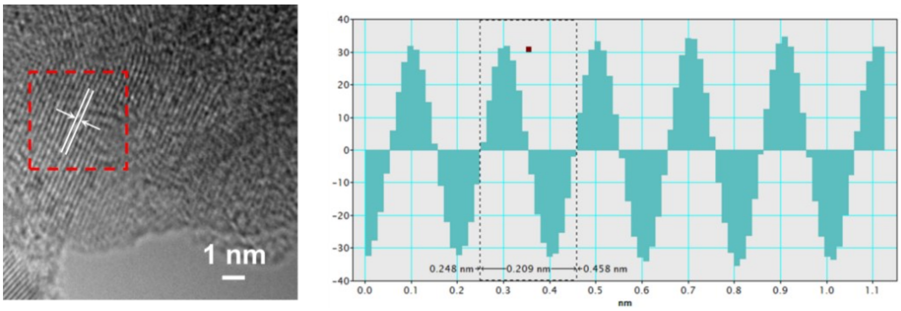

Figure S6. HR-TEM image and intensity profiles measured from the marked in left HR-TEM image of Cu-CP-1-30 electrode.

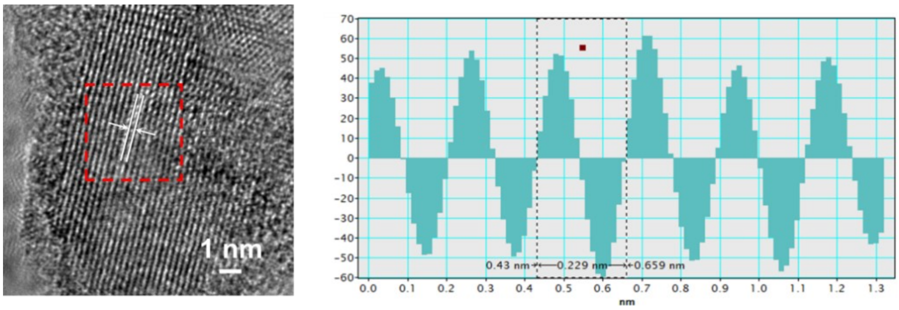

Figure S7. HR-TEM image and intensity profiles measured from the marked in left HR-TEM image of Zn-CP-1-30 electrode.

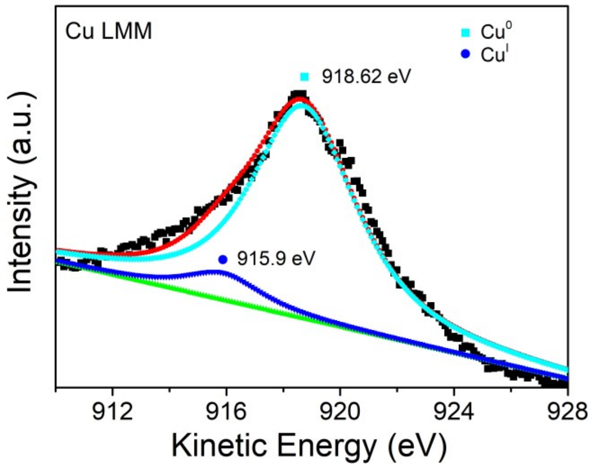

Figure S8. XPS spectra of Auger Cu-LMM spectra of Cu/Zn-CP-1-30 electrode.

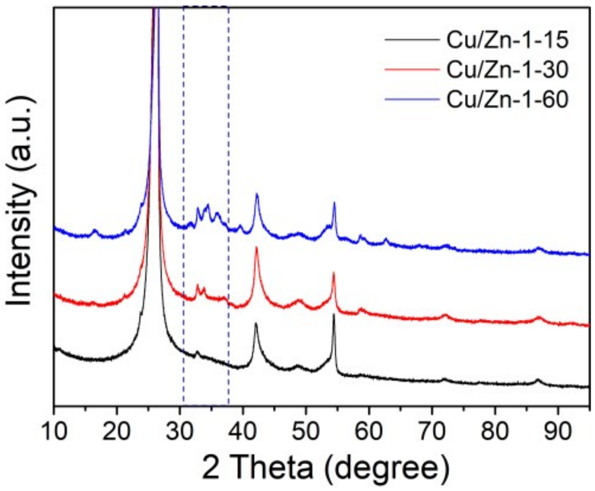

Figure S9. XRD patterns of Cu/Zn-CP-1-15, Cu/Zn-CP-1-30, and Cu/Zn-CP-1-60 electrodes.

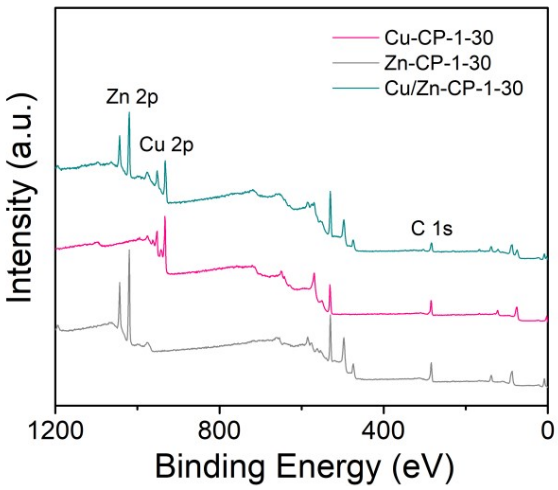

Figure S10. XPS patterns of Cu/Zn-CP-1-15, Cu/Zn-CP-1-30, and Cu/Zn-CP-1-60 electrodes.

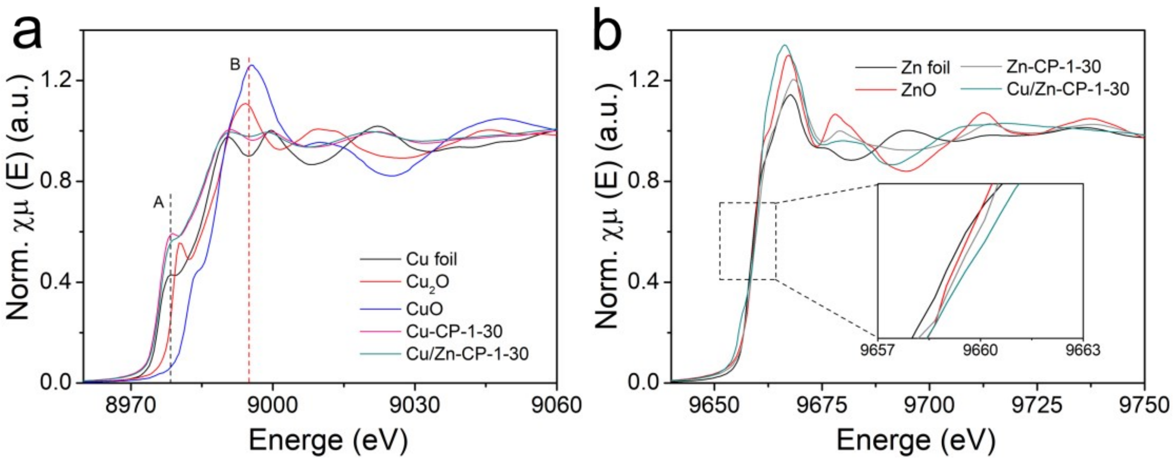

Figure S11. (a) Cu K-edge XANES spectra and (b) Zn K-edge XANES spectra of different electrodes, inset is the partial enlargement around absorption edge. For comparison, reference spectra from Cu foil, Zn foil, Cu_2O, CuO, and ZnO are also shown.

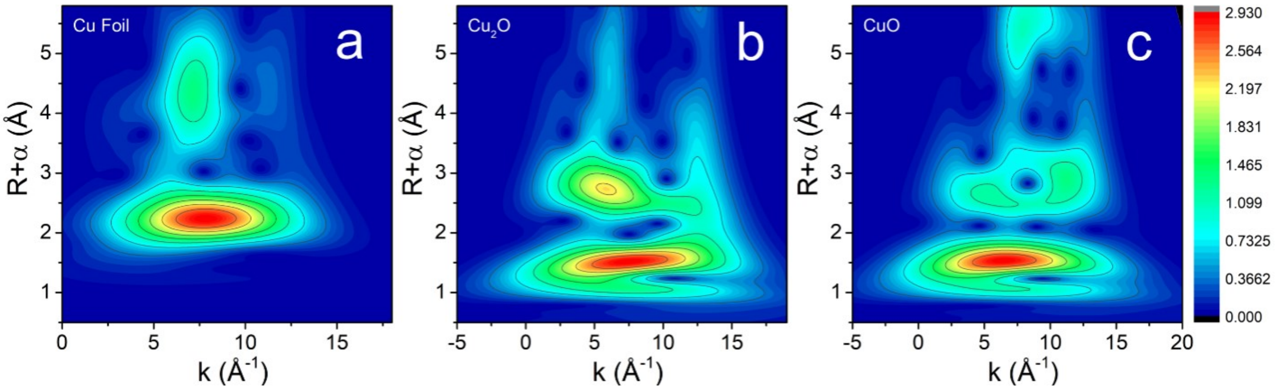

Figure S12. Morlet WT of the k3-weighted Cu-EXAFS data of standard reference samples.

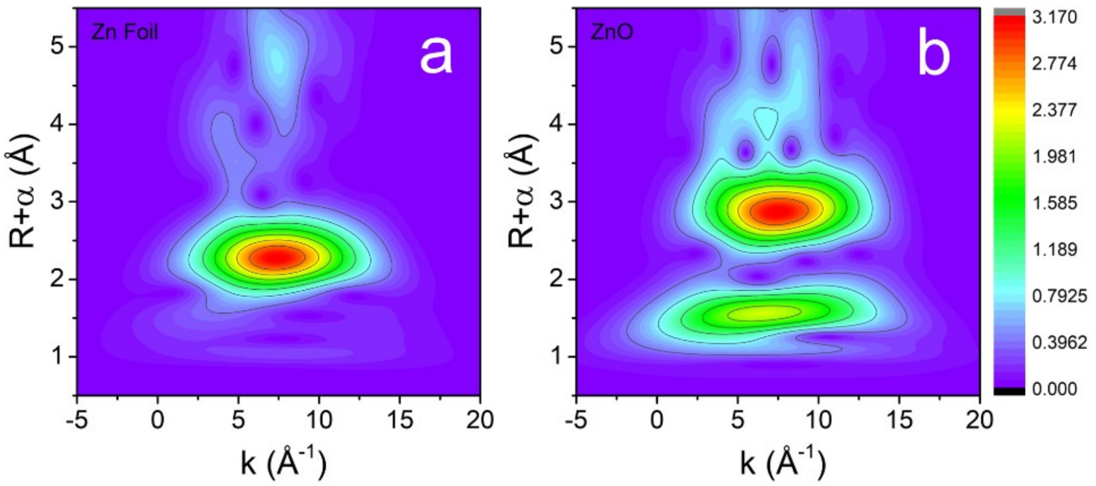

Figure S13. Morlet WT of the k3-weighted Zn-EXAFS spectra of standard reference samples.

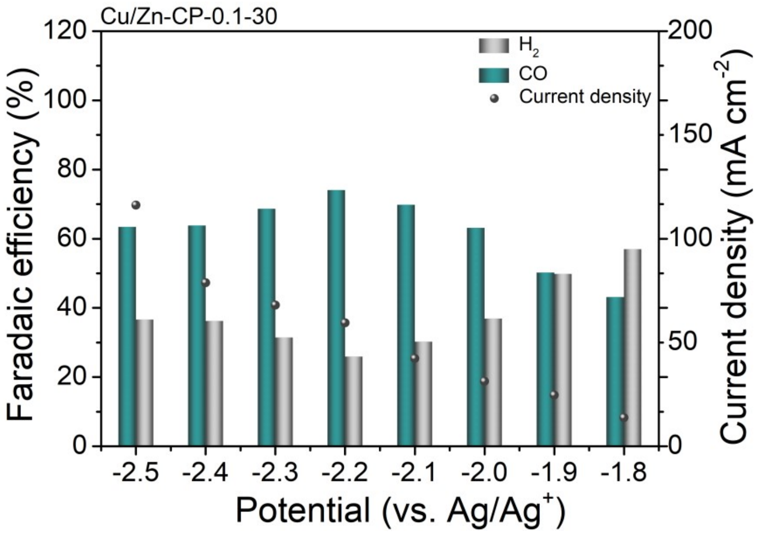

Figure S14. The FE(CO) and current density (*j*) over Cu/Zn-CP-0.1-30 electrode at different applied potentials. Data were obtained at ambient temperature and pressure with a CO_2 stream of 15 sccm.

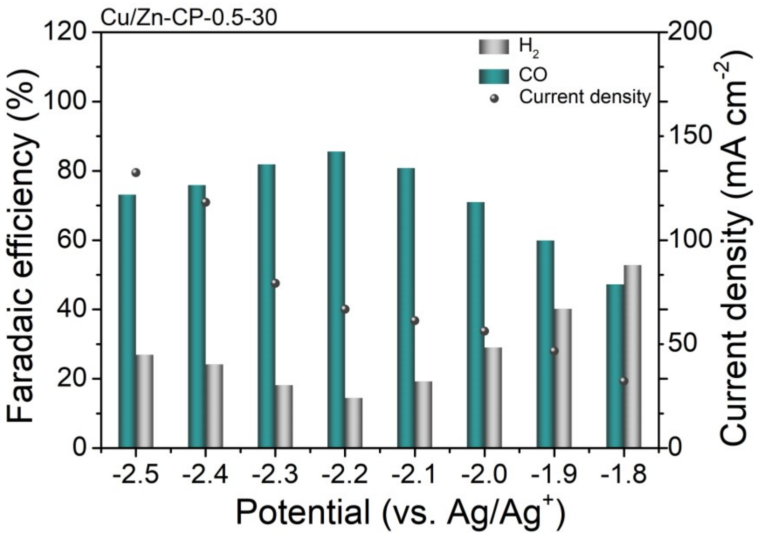

Figure S15. The FE(CO) and current density (*j*) over Cu/Zn-CP-0.5-30 electrode at different applied potentials. Data were obtained at ambient temperature and pressure with a CO_2 stream of 15 sccm.

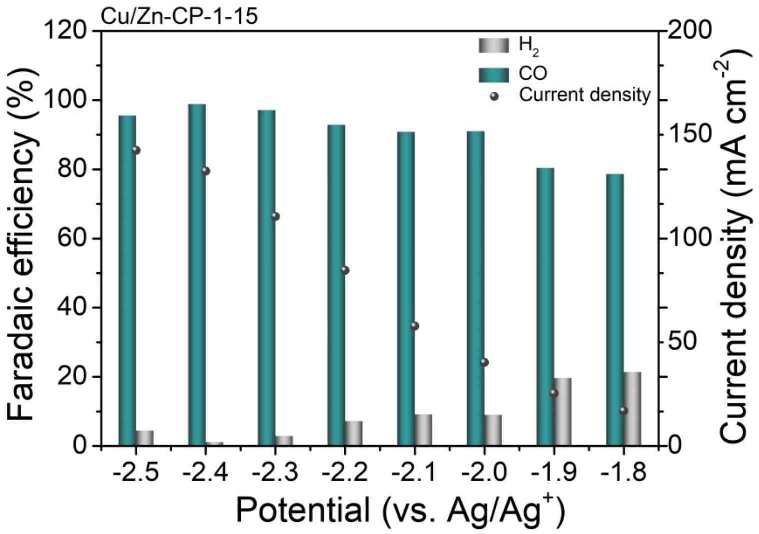

Figure S16. The FE(CO) and current density (*j*) over Cu/Zn-CP-1-15 electrode at different applied potentials. Data were obtained at ambient temperature and pressure with a CO_2 stream of 15 sccm.

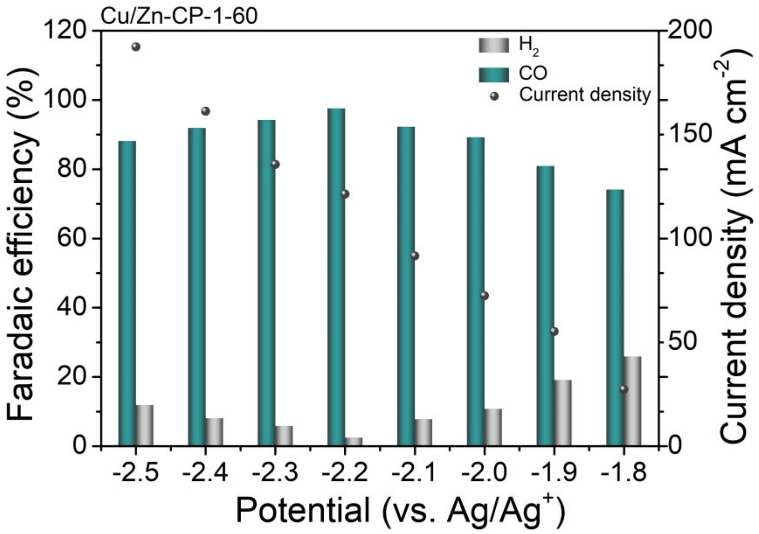

Figure S17. The FE(CO) and current density (*j*) over Cu/Zn-CP-1-60 electrode at different applied potentials. Data were obtained at ambient temperature and pressure with a CO_2 stream of 15 sccm.

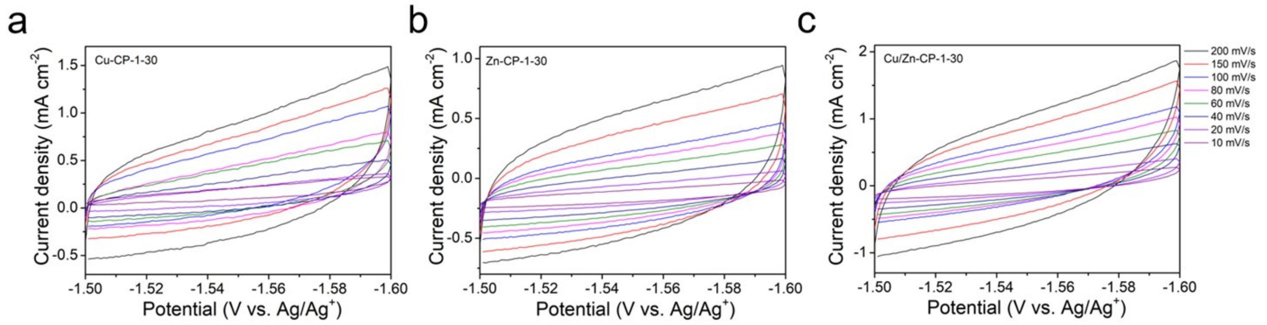

Figure S18. (a-c) Determination of ECSA by measuring the C_{dl} from cyclic voltammetry obtained at various scan rates of Cu-CP-1-30, Zn-CP-1-30, Cu/Zn-CP-1-30 electrodes.

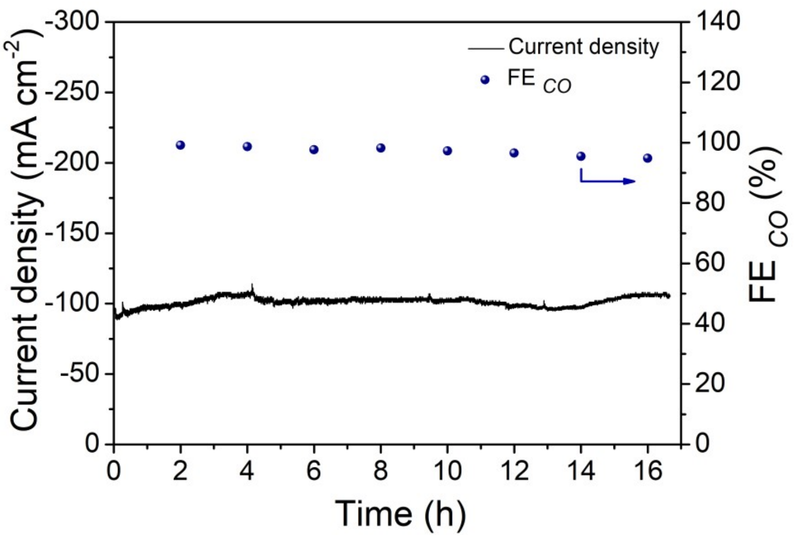

Figure S19. Long-term stability of ECR for Cu/Zn-CP-1-30 electrode at −2.1 V vs. Ag/Ag$^+$.

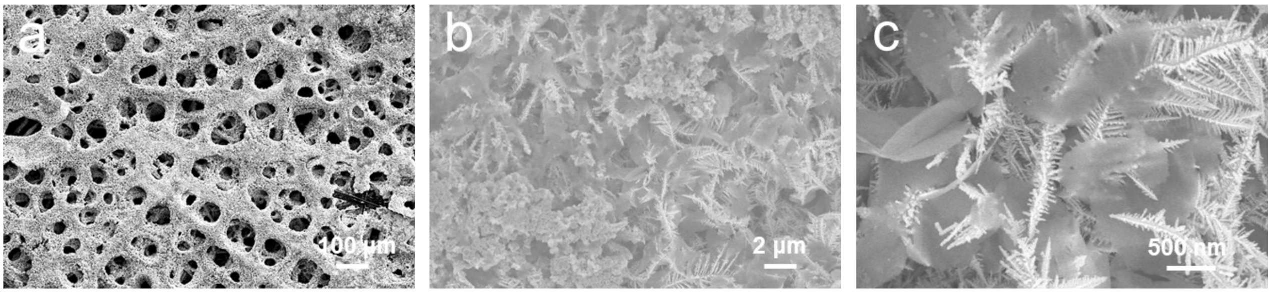

Figure S20. SEM images with a different magnification of Cu/Zn-CP-1-30 electrode after electrolysis in CO_2- saturated 0.5 M [Bmim]PF_6/MeCN electrolyte.

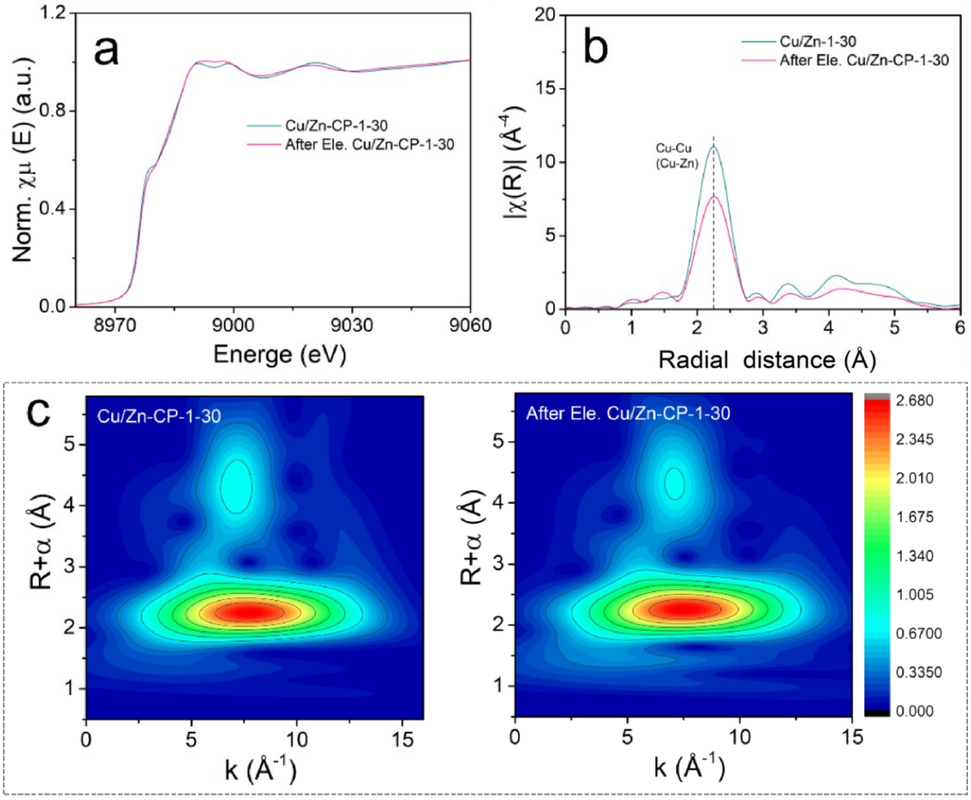

Figure S21. (a) Cu K-edge XANES spectra and Fourier-transformed (FT) k3-weighted (b) Cu K-edge EXAFS data of Cu/Zn-CP-1-30 electrode before and after electrolysis in CO_2-saturated 0.5 M [Bmim]PF_6/MeCN. (c) Morlet WT of the k3-weighted Cu-EXAFS data of Cu/Zn-CP-1-30 electrode before and after electrolysis in CO_2- saturated 0.5 M [Bmim]PF_6/MeCN.

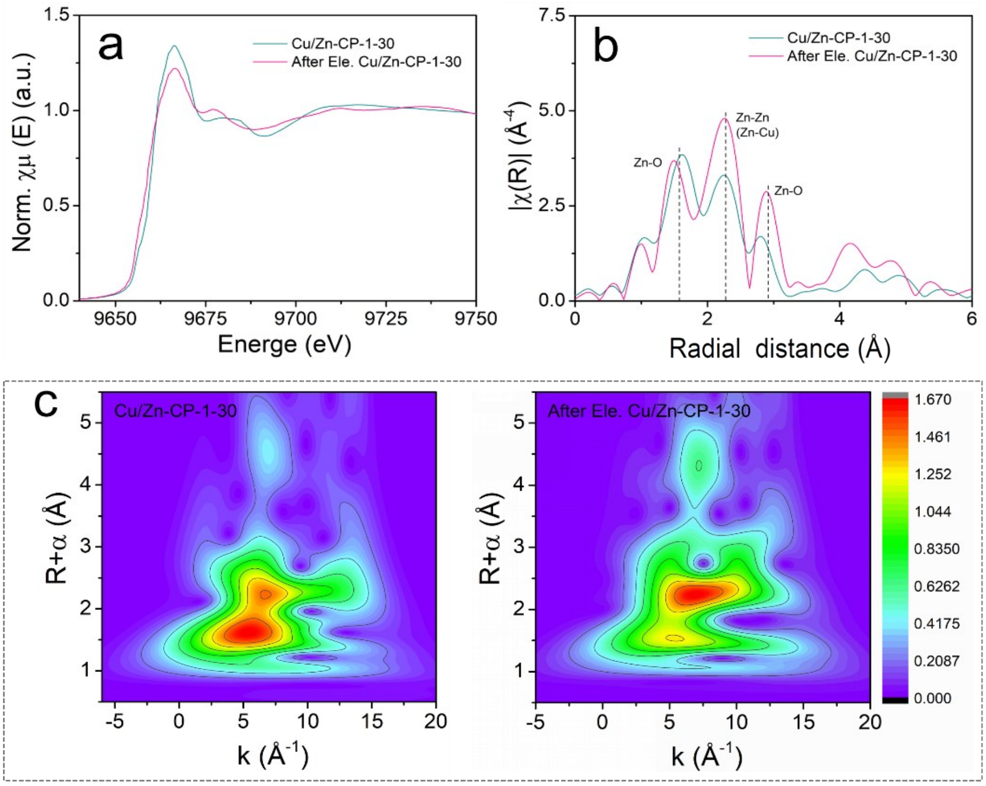

Figure S22. (a) Zn K-edge XANES spectra and Fourier-transformed (FT) k3-weighted (b) Cu K-edge EXAFS data of Cu/Zn-CP-1-30 electrode before and after electrolysis in CO_2-saturated 0.5M [Bmim]PF_6/MeCN. (c) Morlet WT of the k3-weighted Zn-EXAFS spectra of Cu/Zn-CP-1-30 catalysts before and after electrolysis in CO_2- saturated 0.5 M [Bmim]PF_6/MeCN.

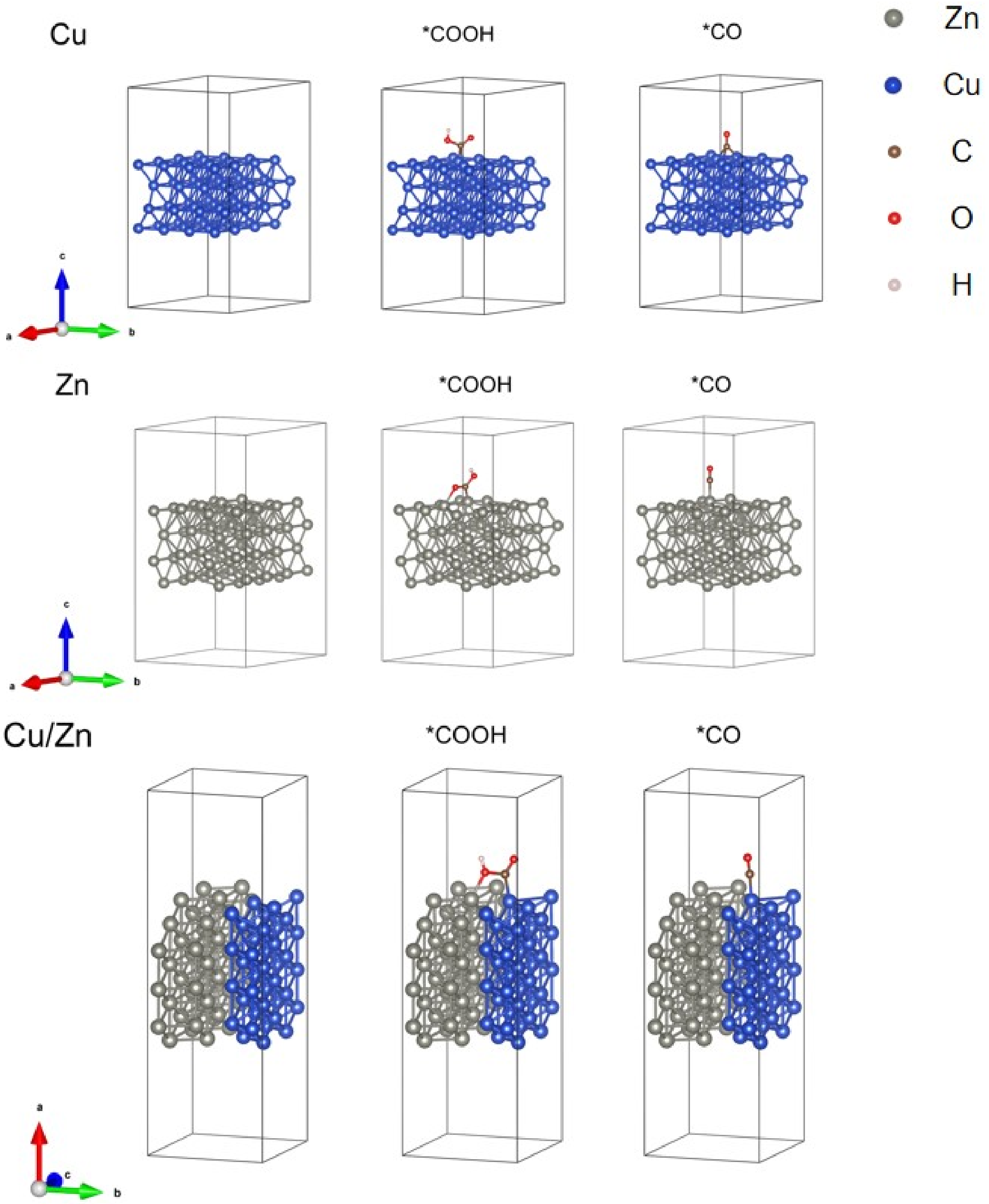

Figure S23. The optimized adsorption configurations of reaction intermediate on the three simulated interface structures.

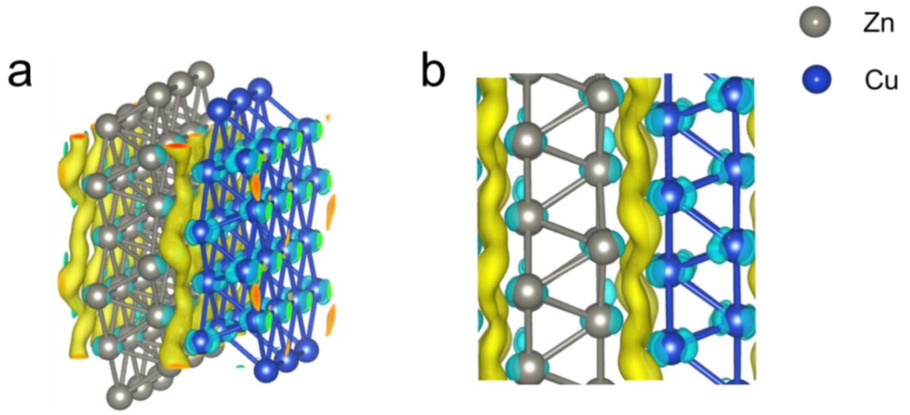

Figure S24. (a-b) Side view of the charge density difference of Cu/Zn hetero-interfaces with an isosurface of $3.6*10^{-3}$ e/Å^3. (The charge accumulation is shown as the yellow region, and the charge depletion is shown as the cyan region.).

Supplementary Tables

Table S1. Comparison of the performance of Cu/Zn-CP-1-30 electrode with some representative ECR to CO reduction catalysts in H-type cell recently reported.

Catalysts	Electrolyte	FE (CO) (%)	J_{CO} (mA cm^{-2})	Reference
Cu/Zn-CP-1-30	0.5 M [Bmim]PF_6/MeCN	99.5	91.8	*This work*
		94.3	165.5	
Cu/In NWs	0.1 M $KHCO_3$	93	4.2	S[7]
Bimetallic Cu-Sn	0.1 M $KHCO_3$	90	3.2	S[8]
Cu-Pd alloy	0.1 M $KHCO_3$	86	6.9	S[9]
Bimetallic Cu/In	0.1 M $KHCO_3$	92	17	S[10]
Cu_1Au_1 SAA	0.1 M $KHCO_3$	91	15.4	S[11]
Zn_1Ni_4-ZIF-8	1.0 M $KHCO_3$	96.5	57.9	S[12]
Core/Shell Cu/SnO_2	0.5 M $KHCO_3$	93	4.6	S[13]
In-Cu bimetallic oxides	0.5 M $KHCO_3$	92.1	11.2	S[14]
Pd-Ag aerogels	0.1 M $NaHCO_3$	84	23	S[15]
ZnO-Ag@UC	0.5 M $KHCO_3$	98.1	22.3	S[16]
AuNi/CNFs	0.1 M $KHCO_3$	92	4	S[17]
Ni/Fe-N-C	0.1 M $KHCO_3$	98	7.4	S[18]
ZnCoNC	0.5 M $KHCO_3$	96.5	26	S[19]
γ-In_2Se_3/CP	30 wt% [Bmim]PF_6/ 65 wt% MeCN/H_2O	96.5	53.4	S[20]
N, P-co-doped carbon aerogels	0.5 M [Bmim]PF_6/MeCN	99.1	143.6	S[21]
Cu-foil	0.3 M $BmimBF_4$/0.2 M $C_{12}mimBF_4$/MeCN	99.5	83.6	S[22]

Table S2. Elemental composition in (w/w) of the Cu and Zn investigated samples determined by ICP-OES.

**Electrodes**	**Cu** $_{(wt\%)}$	**Zn** $_{(wt\%)}$
Cu_1/Zn_5-CP-1-30	13.18	61.49
Cu_1/Zn_2-CP-1-30	33.68	66.32
Cu_1/Zn_1-CP-1-30	51.80	48.20
Cu_2/Zn_1-CP-1-30	72.41	27.59
Cu_5/Zn_1-CP-1-30	66.09	10.65

References

1. G. Kresse, and J. Furthmüller, *Phys. Rev. B,* 1996, **54**, 11169-11186.
2. G. Kresse, and J. Hafner, *Phy. Rev. B,* 1994, **49**, 14251-14269.
3. P. E. Blöchl, *Phys. Rev. B,* 1994, **50**, 17953-17979.
4. Y. Zhang, and W. Yang, *Phys. Rev. Lett.,* 1998, **80**, 890-890.
5. B. Hammer, L. B. Hansen, and J. K. Nørskov, *Phys. Rev. B,* 1999, **59**, 7413-7421.
6. J. K. Nørskov, J. Rossmeisl, A. Logadottir, L. Lindqvist, J. R. Kitchin, T. Bligaard, and H. Jónsson, *J. Phys. Chem. B,* 2004, **108**, 17886-17892.
7. W. Luo, W. Xie, R. Mutschler, E. Oveisi, G. L. De Gregorio, R. Buonsanti, A. Züttel, *ACS Catal.,* 2018, **8**, 6571-6581.
8. S. Sarfraz, A. T. Garcia-Esparza, A. Jedidi, L. Cavallo, K. Takanabe, *ACS Catal.,* 2016, **6**, 2842-2851.
9. Z. Yin, D. Gao, S. Yao, B. Zhao, F. Cai, L. Lin, P. Tang, P. Zhai, G. Wang, D. Ma, X. Bao, *Nano Energy,* 2016, **27**, 35-43.
10. S. Rasul, D. H. Anjum, A. Jedidi, Y. Minenkov, L. Cavallo, K. Takanabe, *Angew. Chem. Int. Ed.,* 2015, **54**, 2146-2150.
11. Y. Zhao, X. Liu, D. Chen, Z. Liu, Q. Yang, X. Lin, M. Peng, P. Liu, Y. Tan, *Sci. China Mater.,* 2021, **64**, 1900-1909.
12. C. Yan, H. Li, Y. Ye, H. Wu, F. Cai, R. Si, J. Xiao, S. Miao, S. Xie, F. Yang, Y. Li, G. Wang, X. Bao, *Energy Environ. Sci.,* 2018, **11**, 1204-1210.
13. Q. Li, J. Fu, W. Zhu, Z. Chen, B. Shen, L. Wu, Z. Xi, T. Wang, G. Lu, J.-j. Zhu, S. Sun, *J. Am. Chem. Soc.,* 2017, **139**, 4290-4293.
14. W. Guo, X. Sun, C. Chen, D. Yang, L. Lu, Y. Yang, B. Han, *Green Chem.,* 2019, **21**, 503-508.
15. Z. Zhang, G. Wen, D. Luo, B. Ren, Y. Zhu, R. Gao, H. Dou, G. Sun, M. Feng, Z. Bai, A. Yu, Z. Chen, *J. Am. Chem. Soc.,* 2021, **143**, 6855-6864.
16. M. Abdinejad, C. Ferrag, M. N. Hossain, M. Noroozifar, K. Kerman, H. B. Kraatz, *J. Mater. Chem. A,* 2021, **9**, 12870-12877.
17. J. Hao, H. Zhu, Y. Li, P. Liu, S. Lu, F. Duan, W. Dong, Y. Lu, T. Liu, M. Du, *Chem. Eng. J.,* 2021, **404**, 126523.
18. W. Ren, X. Tan, W. Yang, C. Jia, S. Xu, K. Wang, S. C. Smith, C. Zhao, *Angew. Chem. Int. Ed.,* 2019, **58**, 6972-6976.
19. W. Zhu, L. Zhang, S. Liu, A. Li, X. Yuan, C. Hu, G. Zhang, W. Deng, K. Zang, J. Luo, Y. Zhu, M. Gu, Z.-J. Zhao, J. Gong, *Angew. Chem. Int. Ed.,* 2020, **59**, 12664-12668.

20. D. Yang, Q. Zhu, X. Sun, C. Chen, W. Guo, G. Yang, B. Han, *Angew. Chem. Int. Ed.,* 2020, **59**, 2354-2359.
21. C. Chen, X. Sun, X. Yan, Y. Wu, H. Liu, Q. Zhu, B. B. A. Bediako, B. Han, *Angew. Chem. Int. Ed.,* 2020, **59**, 11123-11129.
22. J. Yang, X. Kang, J. Jiao, X. Xing, Y. Yin, S. Jia, M. Chu, S. Han, W. Xia, H. Wu, M. He, B. Han, *J. Am. Chem. Soc.,* 2023, **145**, 11512-11517.
